# *Dactylospongia elegans*—A Promising Drug Source: Metabolites, Bioactivities, Biosynthesis, Synthesis, and Structural-Activity Relationship

**DOI:** 10.3390/md20040221

**Published:** 2022-03-23

**Authors:** Sabrin R. M. Ibrahim, Sana A. Fadil, Haifa A. Fadil, Rawan H. Hareeri, Sultan O. Alolayan, Hossam M. Abdallah, Gamal A. Mohamed

**Affiliations:** 1 Department of Chemistry, Preparatory Year Program, Batterjee Medical College, Jeddah 21442, Saudi Arabia; 2 Department of Pharmacognosy, Faculty of Pharmacy, Assiut University, Assiut 71526, Egypt; 3 Department of Natural Products and Alternative Medicine, Faculty of Pharmacy, King Abdulaziz University, Jeddah 21589, Saudi Arabia; safadil@kau.edu.sa (S.A.F.); hmafifi@kau.edu.sa (H.M.A.); gahussein@kau.edu.sa (G.A.M.); 4 Department of Clinical and Hospital Pharmacy, Faculty of Pharmacy, Taibah University, Almadinah Almunawarah 30078, Saudi Arabia; hfadil@taibahu.edu.sa (H.A.F.); solayan@taibahu.edu.sa (S.O.A.); 5 Department of Pharmacology and Toxicology, Faculty of Pharmacy, King Abdulaziz University, Jeddah 21589, Saudi Arabia; rhhareeri@kau.edu.sa; 6 Department of Pharmacognosy, Faculty of Pharmacy, Cairo University, Cairo 11562, Egypt

**Keywords:** sponges, *Dactylospongia elegans*, sesquiterpenes, bioactivities, biosynthesis, synthesis

## Abstract

Marine environment has been identified as a huge reservoir of novel biometabolites that are beneficial for medical treatments, as well as improving human health and well-being. Sponges have been highlighted as one of the most interesting phyla as new metabolites producers. *Dactylospongia elegans* Thiele (Thorectidae) is a wealth pool of various classes of sesquiterpenes, including hydroquinones, quinones, and tetronic acid derivatives. These metabolites possessed a wide array of potent bioactivities such as antitumor, cytotoxicity, antibacterial, and anti-inflammatory. In the current work, the reported metabolites from *D. elegans* have been reviewed, including their bioactivities, biosynthesis, and synthesis, as well as the structural-activity relationship studies. Reviewing the reported studies revealed that these metabolites could contribute to new drug discovery, however, further mechanistic and in vivo studies of these metabolites are needed.

## 1. Introduction

The marine environment is extraordinarily rich in diverse species of organisms that represent an enormous source of biometabolites, many of which have unique chemical entities not present in terrestrial sources [1,2,3]. The molecular diversity and exceptional complexity of marine metabolites have been highlighted in many studies [4,5]. The marine environment investigations have greatly been limited to subtropical and tropical regions, in addition, the exploration has been recently extended to colder regions. However, it is fact that many of these unique marine resources have barely been investigated. 

Sponges (filter feeders, phylum Porifera) are evolutionarily ancient metazoans that occur in marine benthic, quasi-terrestrial, deep-sea, and fresh-water ecosystems [6,7]. They represent one of the important members of marine communities that have potential biotechnological and ecological roles [8,9]. They are sessile multicellular invertebrates, having an enormous amount of tiny pores on their surfaces that allow entrance and circulation of water through canals where organic particles and microorganisms are filtered out and eaten [10]. Calcarea, Demospongiae, Homoscleromorpha, and Hexactinellida are the four main classes of sponges [11]. It is noteworthy that the secondary metabolites distribution among these four different classes of sponges is greatly varied as shown in Table 1.

Sponges are devoid of any physical capacity for defense; therefore, they need to develop specific means and adaptive responses for self-protection [13]. They produce diverse secondary metabolites as defense ways against pathogenic fungi, algae, bacteria, and other predators, also to modulate and/or enable cellular communication [14]. Sponges have been known as a fertile field for the discovery of bioactive metabolites with diverse structural features that have been proven to have a beneficial potential for humans as agricultural medicines, drugs, health foods, and cosmetics [14,15].

Sponges belonging to *Dactylospongia*, particularly *D. elegans* Thiele (Thorectidae), have been vastly recognized as a wealth pool of variable metabolites with a wide array of potent bioactivities. Most of these reported metabolites are sesquiterpenes, including sesquiterpene hydroquinones, sesquiterpene quinones, and sesquiterpene tetronic acids, in addition to few sesterterpenes, sterols, and pregnanes. Further, these metabolites displayed relevant bioactivities, such as antitumor, cytotoxicity, antibacterial, and anti-inflammatory. Diverse studies focusing on the separation, characterization, and bioactivities of *D. elegans* metabolites are reported. Therefore, this works aims to summarize all reported molecules, including their activities, biosynthesis, and synthesis, as well as highlighting the structure–activity relationship studies, which could be used as an extensive reference for further studies on this sponge and its metabolites. Additionally, this work magnifies the relevance of *D. elegans* in the field of marine metabolites production and its significance in the discovery of naturally derived biometabolites. The literature search on *D. elegans* was done by collecting the information on the conducted studies, using scientific databases and websites of various journals, such as Google Scholar, ACS (American Chemical Society), PubMed, Science-Direct, MDPI, Springer Link, Wiley Online Library, Bentham, and Taylor & Francis. 

## 2. Secondary Metabolites of *D. elegans*

Phytochemical studies of *D. elegans* from 1992 until 2022, revealed that 101 metabolites have been separated and characterized from *D. elegans*. These metabolites are grouped according to their chemical classes into sesquiterpenes, sesterterpenes, diterpenes, sterols, pregnanes, and other metabolites which are consequently discussed (Table 2).

**Table 2:** List of reported metabolites from *Dactylospongia elegans*.

The Chemical Structures of Compounds 1–12 (Figure 1), 13–24 (Figure 2), 25–36 (Figure 3), 37–48 (Figure 4), 49–60 (Figure 5), 61–70 (Figure 6), 71–80 (Figure 7), 81–91 (Figure 8), and 92–101 (Figure 9) are illustrated. Compound Name	Extract/Fraction	Mol. Wt.	Mol. Formula	City, Country	Ref.
(−)-Ilimaquinone (**1**)	CH_2_Cl_2_ fraction of MeOH extract	358	C_22_H_30_O_4_	* Similani island, Phuket, Thailand * Papua New Guinea	[16]
	CH_2_Cl_2_ fraction of MeOH extract	-	-	Pulan Tiga, Sabah, Malaysia	[17]
	CH_2_Cl_2_ fraction of MeOH extract	-	-	Pelorus Island, the Great Barrier Reef, Queensland, Australia	[18]
	EtOAc fraction of MeOH extract	-	-	Coral reef of Ishigaki Island, Okinawa, Japan	[19]
	CH_2_Cl_2_ fraction of MeOH extract	-	-	West Flores, Indonesia	[20]
	EtOAc fraction of CH_2_Cl_2_ of MeOH extract	-	-	Coral Gardens dive site at the Inner Gneerings reef, a group of shoals near Mooloolaba, Australia	[21]
	90% and 100% MeOH fraction of RP-18 CC of MeOH extract	-	-	Pugh Shoal, northeast of Truant Island, Australia	[22]
	CH_2_Cl_2_ fraction of H_2_O extract	-	-	* Coast of Malaysia * Coast of Palau	[23]
	CH_2_Cl_2_ fraction of MeOH extract	-	-	West Flores, Indonesia	[24]
	RP-18 CC, 60% MeOH/H_2_O of MeOH extract	-	-	Towo’e Beach Tahuna Bay, Sangihe Islands North Sulawesi, Indonesia	[25]
	CH_2_Cl_2_ fraction of MeOH extract	-	-	Sheraton Caverns, Kauai, Hawaii	[26]
	Et_2_O fraction of acetone extract	-	-	Xisha Island, Hainan, China	[27]
5-(+)*-Epi*-Ilimaquinone (**2**)	CH_2_Cl_2_ fraction of MeOH extract	358	C_22_H_30_O_4_	* Similani island, Phuket, Thailand * Papua New Guinea	[16]
	EtOAc fraction of MeOH extract	-	-	Coral reef of Ishigaki Island, Okinawa, Japan	[19]
	CH_2_Cl_2_ fraction of MeOH extract	-	-	West Flores, Indonesia	[20]
	90% and 100% MeOH fraction of RP-18 CC/MeOH extract	-	-	Pugh Shoal, northeast of Truant Island, Australia	[22]
	*n*-Hexane fraction of MeOH extract	-	-	Island of Ambon, Indonesia	[28]
	CH_2_Cl_2_ fraction of MeOH extract	-	-	Sheraton Caverns, Kauai, Hawaii	[26]
	Et_2_O fraction of acetone extract	-	-	Xisha Island, Hainan, China	[27]
(−)-5,8-*Diepi*-Ilimaquinone (**3**)	CH_2_Cl_2_ fraction of H_2_O extract	358	C_22_H_30_O_4_	* Coast of Malaysia * Coast of Palau	[23]
(−)-Dactyloquinone A (**4**)	EtOAc fraction of MeOH extract	356	C_22_H_28_O_4_	Coral reef of Ishigaki Island, Okinawa, Japan	[29]
	EtOAc fraction of MeOH extract	-	-	Coral reef of Ishigaki Island, Okinawa, Japan	[19]
	Et_2_O fraction of acetone extract	-	-	Xisha Island, Hainan, China	[27]
(−)-Dactyloquinone B (**5**)	EtOAc fraction of MeOH extract	356	C_22_H_28_O_4_	Coral reef of Ishigaki Island, Okinawa, Japan	[29]
	EtOAc fraction of MeOH extract	-	-	Coral reef of Ishigaki Island, Okinawa, Japan	[19]
	90% and 100% MeOH fraction of RP-18 CC of MeOH extract	-	-	Pugh Shoal, northeast of Truant Island, Australia	[22]
	Et_2_O fraction of acetone extract	-	-	Xisha Island, Hainan, China	[27]
(+)-8-*Epi*-Dactyloquinone B (**6**)	CH_2_Cl_2_ fraction of H_2_O extract	356	C_22_H_28_O_4_	* Coast of Malaysia * Coast of Palau	[23]
(−)-Dactyloquinone C (**7**)	EtOAc fraction of MeOH extract	356	C_22_H_28_O_4_	Coral reef of Ishigaki Island, Okinawa, Japan	[19]
	Et_2_O fraction of acetone extract	-	-	Xisha Island, Hainan, China	[27]
(−)-Dactyloquinone D (**8**)	EtOAc fraction of MeOH extract	356	C_22_H_28_O_4_	Coral reef of Ishigaki Island, Okinawa, Japan	[19]
	Et_2_O fraction of acetone extract	-	-	Xisha Island, Hainan, China	[27]
(+)-Dactyloquinone E (**9**)	EtOAc fraction of MeOH extract	356	C_22_H_28_O_4_	Coral reef of Ishigaki Island, Okinawa, Japan	[19]
	Et_2_O fraction of acetone extract	-	-	Xisha Island, Hainan, China	[27]
(+)-Neodactyloquinone (**10**)	EtOAc fraction of MeOH extract	356	C_22_H_28_O_4_	Coral reef of Ishigaki Island, Okinawa, Japan	[30]
(+)-Isospongiaquinone (**11**)	CH_2_Cl_2_ fraction of MeOH extract	358	C_22_H_30_O_4_	* Similani island, Phuket, Thailand * Papua New Guinea	[16]
	*n*-Hexane fraction of MeOH extract	-	-	Island of Ambon, Indonesia	[28]
Bolinaquinone (**12**)	CH_2_Cl_2_ fraction of MeOH extract	358	C_22_H_30_O_4_	West Flores, Indonesia	[24]
Dictyoceratidaquinone (**13**)	EtOAc fraction of CH_2_Cl_2_ of MeOH extract	358	C_22_H_30_O_4_	Coral Gardens dive site at the Inner Gneerings reef, a group of shoals near Mooloolaba, Australia	[21]
Mamanuthaquinone (**14**)	EtOAc fraction of CH_2_Cl_2_ of MeOH extract	358	C_22_H_30_O_4_	Coral Gardens dive site at the Inner Gneerings reef, a group of shoals near Mooloolaba (Australia),	[21]
Hyatellaquinone (**15**)	EtOAc fraction of CH_2_Cl_2_ of MeOH extract	358	C_22_H_30_O_4_	Coral Gardens dive site at the Inner Gneerings reef, a group of shoals near Mooloolaba, Australia	[21]
(+)-Isohyatellaquinone (**16**)	EtOAc fraction of CH_2_Cl_2_ of MeOH extract	358	C_22_H_30_O_4_	Coral Gardens dive site at the Inner Gneerings reef, a group of shoals near Mooloolaba, Australia	[21]
(−)-*Ent*-Isohyatellaquinone (**17**)	EtOAc fraction of CH_2_Cl_2_ of MeOH extract	358	C_22_H_30_O_4_	Coral Gardens dive site at the Inner Gneerings reef, a group of shoals near Mooloolaba, Australia	[21]
Neomamanuthaquinone (**18**)	EtOAc fraction of CH_2_Cl_2_ of MeOH extract	344	C_21_H_28_O_4_	Coral Gardens dive site at the Inner Gneerings reef, a group of shoals near Mooloolaba, Australia	[21]
7,8-Dehydrocyclospongiaquinone-2 (**19**)	EtOAc fraction of CH_2_Cl_2_ of MeOH extract	356	C_22_H_28_O_4_	Coral Gardens dive site at the Inner Gneerings reef, a group of shoals near Mooloolaba, Australia	[21]
9-*Epi*-7,8-Dehydrocyclospongiaquinone-2 (**20**)	EtOAc fraction of CH_2_Cl_2_ of MeOH extract	356	C_22_H_28_O_4_	Coral Gardens dive site at the Inner Gneerings reef, a group of shoals near Mooloolaba, Australia	[21]
Cyclospongiaquinone-1 (**21**)	CH_2_Cl_2_ fraction of H_2_O extract	358	C_22_H_30_O_4_	* Coast of Malaysia * Coast of Palau	[23]
Cyclospongiaquinone-2 (**22**)	CH_2_Cl_2_ fraction of H_2_O extract	358	C_22_H_30_O_4_	* Coast of Malaysia * Coast of Palau	[23]
(−)-4,5-*Diepi*-Dactylospongiaquinone (**23**)	CH_2_Cl_2_ fraction of H_2_O extract	358	C_22_H_30_O_4_	* Coast of Malaysia * Coast of Palau	[23]
(−)-10,17-*O*-Cyclo-4,5-*diepi*-dactylospongiaquinone (**24**)	CH_2_Cl_2_ fraction of H_2_O extract	356	C_22_H_28_O_4_	* Coast of Malaysia * Coast of Palau	[23]
Smenospongine (**25**)	CH_2_Cl_2_ fraction of MeOH extract	343	C_21_H_29_NO_3_	* Similani island, Phuket, Thailand * Papua New Guinea	[16]
	CH_2_Cl_2_ fraction of MeOH extract	-	-	West Flores, Indonesia	[20]
	CH_2_Cl_2_ fraction of MeOH extract	-	-	West Flores, Indonesia	[31]
	CH_2_Cl_2_ fraction of MeOH extract	-	-	West Flores, Indonesia	[24]
	RP-18 CC, 60% MeOH/H_2_O of MeOH extract	-	-	Towo’e Beach Tahuna Bay, Sangihe Islands North Sulawesi, Indonesia	[25]
	CH_2_Cl_2_ fraction of MeOH extract	-	-	Sheraton Caverns, Kauai, Hawaii	[26]
5-(+)-*Epi*-Smenospongine (**26**)	CH_2_Cl_2_ fraction of MeOH extract	343	C_21_H_29_NO_3_	West Flores, Indonesia	[20]
	CH_2_Cl_2_ fraction of MeOH extract	-	-	West Flores, Indonesia	[24]
	*n*-Hexane fraction of MeOH extract	-	-	Island of Ambon, Indonesia	[28]
Smenospongimine (**27**)	CH_2_Cl_2_/MeOH fractions of EtOH extract	357	C_22_H_31_NO_3_	Yongxing Island, South China Sea	[32]
Smenospongine B (**28**)	90% and 100% MeOH fraction of RP-18 CC of MeOH extract	401	C_23_H_31_NO_5_	Pugh Shoal, northeast of Truant Island, Australia	[22]
Smenospongine C (**29**)	90% and 100% MeOH fraction of RP-18 CC of MeOH extract	415	C_24_H_33_NO_5_	Pugh Shoal, northeast of Truant Island, Australia	[22]
	*n*-Hexane fraction of MeOH extract	-	-	Island of Ambon, Indonesia	[28]
Smenospongorine (**30**)	CH_2_Cl_2_ fraction of MeOH extract	399	C_25_H_37_NO_3_	West Flores, Indonesia	[20]
	*n*-Hexane fraction of MeOH extract	-	-	Sheraton Caverns, Kauai, Hawaii	[26]
	CH_2_Cl_2_/MeOH fraction of EtOH extract	-	-	Yongxing Island, South China Sea	[32]
5-(+)-*Epi*-Smenospongorine (**31**)	CH_2_Cl_2_ fraction of MeOH extract	399	C_25_H_37_NO_3_	West Flores, Indonesia	[20]
	CH_2_Cl_2_ fraction of MeOH extract	-	-	West Flores, Indonesia	[24]
	CH_2_Cl_2_ fraction of MeOH extract	-	-	West Flores, Indonesia	[24]
Smenospongiarine (**32**)	CH_2_Cl_2_ fraction of MeOH extract	413	C_26_H_39_NO_3_	* Similani island, Phuket, Thailand * Papua New Guinea	[16]
	CH_2_Cl_2_/MeOH fraction of EtOH extract	-	-	Yongxing Island, South China Sea	[32]
5-(+)-*Epi*-Smenospongiarine (**33**)	CH_2_Cl_2_ fraction of MeOH extract	413	C_26_H_39_NO_3_	* Similani island, Phuket, Thailand * Papua New Guinea	[15]
	*n*-Hexane fraction of MeOH extract	-	-	Sheraton Caverns, Kauai, Hawaii	[26]
Nakijiquinone D (34)	*n*-Hexane fraction of MeOH extract	445	C_25_H_35_NO_6_	Island of Ambon, Indonesia	[28]
Smenospongidine (**35**)	CH_2_Cl_2_ fraction of MeOH extract	447	C_29_H_37_NO_3_	* Similani island, Phuket, Thailand * Papua New Guinea	[16]
	CH_2_Cl_2_ fraction of MeOH extract	-	-	West Flores, Indonesia	[20]
	CH_2_Cl_2_ fraction of MeOH extract	-	-	Sheraton Caverns, Kauai, Hawaii	[26]
5-(+)*-Epi*-Smenospongidine (**36**)	CH_2_Cl_2_ fraction of MeOH extract	447	C_29_H_37_NO_3_	* Similani island, Phuket, Thailand * Papua New Guinea	[16]
	CH_2_Cl_2_ fraction of MeOH extract	-	-	West Flores, Indonesia	[20]
	CH_2_Cl_2_ fraction of MeOH extract	-	-	West Flores, Indonesia	[24]
	*n*-Hexane fraction of MeOH extract	-	-	Island of Ambon, Indonesia	[28]
Nakijiquinone V (**37**)	RP-18 CC, 60% MeOH/H_2_O of MeOH extract	437	C_26_H_35_N_3_O_3_	Towo’e Beach Tahuna Bay, Sangihe Islands, North Sulawesi, Indonesia	[25]
Dysideamine (**38**)	CH_2_Cl_2_ fraction of MeOH extract	343	C_21_H_29_NO_3_	West Flores, Indonesia	[24]
Isosmenospongine (**39**)	*n*-Hexane fraction of MeOH extract	343	C_21_H_29_NO_3_	Island of Ambon, Indonesia	[28]
Nakijiquinone A (**40**)	*n*-Hexane fraction of MeOH extract	401	C_23_H_31_NO_5_	Island of Ambon, Indonesia	[28]
Nakijiquinone B (**41**)	*n*-Hexane fraction of MeOH extract	443	C_26_H_37_NO_5_	Island of Ambon, Indonesia	[28]
Nakijiquinone C (**42**)	*n*-Hexane fraction of MeOH extract	431	C_24_H_33_NO_6_	Island of Ambon, Indonesia	[28]
Nakijiquinone G (**43**)	*n*-Hexane fraction of MeOH extract	437	C_26_H_35_N_3_O_3_	Island of Ambon, Indonesia	[28]
5-*Epi*-Nakijiquinone Q (**44**)	*n*-Hexane fraction of MeOH extract	447	C_29_H_37_NO_3_	Island of Ambon, Indonesia	[28]
20-Demethoxy-20-methylaminodactyloquinone D (**45**)	CH_2_Cl_2_/MeOH fraction of EtOH extract	355	C_22_H_29_NO_3_	Yongxing Island, South China Sea	[32]
20-Demethoxy-20-isobutylaminodactyloquinone D (**46**)	CH_2_Cl_2_/MeOH fraction of EtOH extract	397	C_25_H_35_NO_3_	Yongxing Island, South China Sea	[32]
20-Demethoxy-20-isopentylaminodactyloquinone D (**47**)	CH_2_Cl_2_/MeOH fraction of EtOH extract	411	C_26_H_37_NO_3_	Yongxing Island, South China Sea	[32]
(+)-Smenospondiol (**48**)	CH_2_Cl_2_ fraction of MeOH extract	372	C_23_H_32_O_4_	* Similani island, Phuket, Thailand * Papua New Guinea	[16]
	CH_2_Cl_2_ fraction of MeOH extract	-	-	West Flores, Indonesia	[20]
	CH_2_Cl_2_ fraction of MeOH extract	-	-	West Flores, Indonesia	[24]
(+)-Dictyoceratin A (**49**)	CH_2_Cl_2_ fraction of MeOH extract	372	C_23_H_32_O_4_	* Similani island, Phuket, Thailand * Papua New Guinea	[16]
	*n*-Hexane fraction of MeOH extract	-	-	Sheraton Caverns, Kauai, Hawaii	[26]
	Et_2_O fraction of acetone extract	-	-	Xisha Island, Hainan, China	[27]
19-Methoxy-dictyoceratin-A (**50**)	CH_2_Cl_2_/MeOH fractions of EtOH extract	402	C_24_H_34_O_5_	Yongxing Island, South China Sea	[32]
(+)-Dictyoceratin B (**51**)	*n*-Hexane fraction of MeOH extract	388	C_23_H_32_O_5_	Sheraton Caverns, Kauai, Hawaii	[26]
	Et_2_O fraction of acetone extract	-	-	Xisha Island, Hainan, China	[27]
(+)-Dictyoceratin C (**52**)	CH_2_Cl_2_ fraction of MeOH extract	356	C_23_H_32_O_3_	Pulan Tiga, Sabah, Malaysia	[17]
	CH_2_Cl_2_ fraction of MeOH extract	-	-	West Flores, Indonesia	[20]
	CH_2_Cl_2_ fraction of MeOH extract	-	-	West Flores, Indonesia	[24]
	EtOAc fraction of EtOH extract	-	-	Meishan coral reef, Sanya, China	[33]
	RP-18 CC, 60% MeOH/H_2_O of MeOH extract	-	-	Towo’e Beach Tahuna Bay, Sangihe Islands North Sulawesi, Indonesia	[25]
	*n*-Hexane fraction of MeOH extract	-	-	Sheraton Caverns, Kauai, Hawaii	[26]
	CH_2_Cl_2_/MeOH fraction of EtOH extract	-	-	Yongxing Island, South China Sea	[32]
	Et_2_O fraction of acetone extract	-	-	Xisha Island, Hainan, China	[27]
Polyfibrospongol A (**53**)	EtOAc fraction of EtOH extract	386	C_24_H_34_O_4_	Meishan coral reef, Sanya, China	[33]
	Et_2_O fraction of acetone extract	-	-	Xisha Island, Hainan, China	[27]
(−)-Xishaeleganin C (**54**)	Et_2_O fraction of acetone extract	372	C_23_H_32_O_4_	Xisha Island, Hainan, China	[27]
(+)-Xishaeleganin D (**55**)	Et_2_O fraction of acetone extract	356	C_23_H_32_O_3_	Xisha Island, Hainan, China	[27]
(−)-Xishaeleganin A (**56**)	Et_2_O fraction of acetone extract	386	C_24_H_34_O_4_	Xisha Island, Hainan, China	[27]
(−)-Xishaeleganin B (**57**)	Et_2_O fraction of acetone extract	390	C_23_H_34_O_5_	Xisha Island, Hainan, China	[27]
(−)-Smenodiol (**58**)	CH_2_Cl_2_ fraction of MeOH extract	372	C_23_H_32_O_4_	* Similani island, Phuket, Thailand * Papua New Guinea	[16]
	CH_2_Cl_2_ fraction of MeOH extract	-	-	Pelorus Island, the Great Barrier Reef, Queensland, Australia	[18]
(−)-Dactylosponol (**59**)	CH_2_Cl_2_ fraction of MeOH extract	356	C_23_H_32_O_3_	* Similani island, Phuket, Thailand * Papua New Guinea	[16]
(−)-Dactylospontriol (**60**)	CH_2_Cl_2_ fraction of MeOH extract	388	C_23_H_32_O_5_	* Similani island, Phuket, Thailand * Papua New Guinea	[16]
(+)-Cyclospongiacatechol (**61**)	CH_2_Cl_2_ fraction of H_2_O extract	388	C_23_H_32_O_5_	* Coast of Malaysia * Coast of Palau	[23]
Chromazonarol (**62**)	CH_2_Cl_2_ fraction of MeOH extract	314	C_21_H_30_O_2_	* Similani island, Phuket, Thailand * Papua New Guinea	[16]
8-*Epi*-Chromazonarol (**63**)	CH_2_Cl_2_ fraction of MeOH extract	314	C_21_H_30_O_2_	* Similani island, Phuket, Thailand * Papua New Guinea	[16]
	CH_2_Cl_2_ fraction of H_2_O extract	-	-	* Coast of Malaysia * Coast of Palau	[23]
Pelorol (**64**)	CH_2_Cl_2_ fraction of MeOH extract	372	C_23_H_32_O_4_	Pelorus Island, the Great Barrier Reef, Queensland, Australia	[18]
	*n*-Hexane fraction of MeOH extract	-	-	Island of Ambon, Indonesia	[28]
Nakijinol B (**65**)	90% and 100% MeOH fraction of RP-18 CC of MeOH extract	355	C_22_H_29_NO_3_	Pugh Shoal, northeast of Truant Island, Australia	[22]
Popolohuanone B (**66**)	CH_2_Cl_2_ fraction of CH_2_Cl_2_/MeOH extract	623	C_42_H_57_NO_3_	Xisha Islands maritime space, South China Sea	[34]
Popolohuanone C (**67**)	CH_2_Cl_2_ fraction of CH_2_Cl_2_/MeOH extract	623	C_42_H_57_NO_3_	Xisha Islands maritime space, South China Sea	[34]
Popolohuanone G (**68**)	CH_2_Cl_2_ fraction of CH_2_Cl_2_/MeOH extract	642	C_42_H_56_O_4_	Xisha Islands maritime space, South China Sea	[34]
Popolohuanone H (**69**)	CH_2_Cl_2_ fraction of CH_2_Cl_2_/MeOH extract	623	C_42_H_57_NO_3_	Xisha Islands maritime space, South China Sea	[34]
Popolohuanone I (**70**)	CH_2_Cl_2_ fraction of CH_2_Cl_2_/MeOH extract	623	C_42_H_57_NO_3_	Xisha Islands maritime space, South China Sea	[34]
(−)-Dactyltronic acid A (**71**)	CH_2_Cl_2_ fraction of MeOH extract	362	C_21_H_30_O_5_	Pulan Tiga, Sabah, Malaysia	[17]
	EtOAc fraction of EtOH extract	-	-	Meishan coral reef, Sanya, China	[33]
(−)-Dactyltronic acid B (**72**)	CH_2_Cl_2_ fraction of MeOH extract	362	C_21_H_30_O_5_	Pulan Tiga, Sabah, Malaysia	[17]
	EtOAc fraction of EtOH extract	-	-	Meishan coral reef, Sanya, China	[33]
(+)-Dactylolactone A (**73**)	EtOAc fraction of MeOH extract	404	C_23_H_32_O_6_	Coral reef of Ishigaki Island, Okinawa, Japan	[30]
(+)-Dactylolactone B (**74**)	EtOAc fraction of MeOH extract	404	C_23_H_32_O_6_	Coral reef of Ishigaki Island, Okinawa, Japan	[30]
(−)-Dactylolactone C (**75**)	EtOAc fraction of MeOH extract	404	C_23_H_32_O_6_	Coral reef of Ishigaki Island, Okinawa, Japan	[30]
(−)-Dactylolactone D (**76**)	EtOAc fraction of MeOH extract	404	C_23_H_32_O_6_	Coral reef of Ishigaki Island, Okinawa, Japan	[30]
(+)-Dactylospene B (**77**)	CH_2_Cl_2_/MeOH fraction of EtOH extract	400	C_26_H_40_O_3_	Yongxing Island, South China Sea	[35]
(+)-Dactylospene C (**78**)	CH_2_Cl_2_/MeOH fraction of EtOH extract	400	C_26_H_40_O_3_	Yongxing Island, South China Sea	[35]
(+)-Dactylospene D (**79**)	CH_2_Cl_2_/MeOH fraction of EtOH extract	432	C_27_H_44_O_4_	Yongxing Island, South China Sea	[35]
(+)-Dactylospene E (**80**)	CH_2_Cl_2_/MeOH fraction of EtOH extract	432	C_27_H_44_O_4_	Yongxing Island, South China Sea	[35]
Dactylospongenone A (**81**)	CH_2_Cl_2_ fraction of MeOH extract	390	C_23_H_34_O_5_	* Similani island, Phuket, Thailand * Papua New Guinea	[16]
	CH_2_Cl_2_ fraction of MeOH extract	-	-	Pulan Tiga, Sabah, Malaysia	[17]
Dactylospongenone B (**82**)	CH_2_Cl_2_ fraction of MeOH extract	390	C_23_H_34_O_5_	* Similani island, Phuket, Thailand * Papua New Guinea	[16]
	CH_2_Cl_2_ fraction of MeOH extract	-	-	Pulan Tiga, Sabah, Malaysia	[17]
Dactylospongenone C (**83**)	CH_2_Cl_2_ fraction of MeOH extract	390	C_23_H_34_O_5_	* Similani island, Phuket, Thailand * Papua New Guinea	[16]
	CH_2_Cl_2_ fraction of MeOH extract	-	-	Pulan Tiga, Sabah, Malaysia	[17]
Dactylospongenone D (**84**)	CH_2_Cl_2_ fraction of MeOH extract	390	C_23_H_34_O_5_	* Similani island, Phuket, Thailand * Papua New Guinea	[16]
	CH_2_Cl_2_ fraction of MeOH extract	-	-	Pulan Tiga, Sabah, Malaysia	[17]
Dactylospongenone G (**85**)	*n*-Hexane fraction of MeOH extract	404	C_23_H_32_O_6_	Island of Ambon, Indonesia	[28]
Dactylospongenone H (**86**)	*n*-Hexane fraction of MeOH extract	390	C_23_H_34_O_5_	Island of Ambon, Indonesia	[28]
(−)-Smenospongic acid (**87**)	CH_2_Cl_2_ fraction of MeOH extract	250	C_16_H_26_O_2_	* Similani island, Phuket, Thailand * Papua New Guinea	[16]
	CH_2_Cl_2_ fraction of MeOH extract	-	-	Pulan Tiga, Sabah, Malaysia	[17]
(+)-Eleganstone A (**88**)	CH_2_Cl_2_ fraction of EtOH extract	404	C_24_H_36_O_5_	Yongxing Island and Seven Connected Islets, South China Sea	[36]
Diacetoxydolabella-2,7-dien-6-one (**89**)	CH_2_Cl_2_ fraction of EtOH extract	404	C_24_H_36_O_5_	Yongxing Island and Seven Connected Islets, South China Sea	[36]
(+)-(1R*,2E,4R*,8E,10S*,11S*,12R*)-10,18-Diacetoxydolabella-2,8-dien-6-one (**90**)	CH_2_Cl_2_ fraction of EtOH extract	404	C_24_H_36_O_5_	Yongxing Island and Seven Connected Islets, South China Sea	[36]
(1R*,2E,4R*,7Z,10S*,11S*,12R*)-10,18-Diacetoxydolabella-2,7-dien- 6-one (**91**)	CH_2_Cl_2_ fraction of EtOH extract	404	C_24_H_36_O_5_	Yongxing Island and Seven Connected Islets, South China Sea	[36]
Furospinosulin-1 (**92**)	EtOAc fraction of CH_2_Cl_2_ of MeOH extract	354	C_25_H_38_O	Coral Gardens dive site at the Inner Gneerings reef, a group of shoals near Mooloolaba, Australia	[21]
Furospinosulin B (**93**)	CH_2_Cl_2_/MeOH fractions of EtOH extract	370	C_25_H_38_O_2_	Yongxing Island, South China Sea	[35]
(−)-Luffariellolide (**94**)	CH_2_Cl_2_/MeOH fractions of EtOH extract	386	C_25_H_38_O_3_	Yongxing Island, South China Sea	[35]
(−)-Dactylospene A (**95**)	CH_2_Cl_2_/MeOH fractions of EtOH extract	386	C_25_H_38_O_3_	Yongxing Island, South China Sea	[35]
Pregna-1,20-dien-3-one (**96**)	EtOAc fraction of EtOH extract	298	C_21_H_30_O	Meishan coral reef, Sanya, China	[33]
3-Hydroxycholesta-5,8-dien-7-one (**97**)	EtOAc fraction of EtOH extract	398	C_27_H_42_O_2_	Meishan coral reef, Sanya, China	[33]
(3S,5R,9R,10S,13R,17R,20R,24S,22E)-Ergosta-6,8,22-triene-3,25-diol (**98**)	CH_2_Cl_2_ fraction of CH_2_Cl_2_/MeOH extract	412	C_28_H_44_O_2_	Xisha islands maritime space, South China Sea	[37]
(3S,5R,9R,10S,13R,17R,20R,24S,22E)-Ergosta-6,8,22-triene-25-ol-3-sulfonate (**99**)	CH_2_Cl_2_ fraction of CH_2_Cl_2_/MeOH extract	492	C_28_H_44_O_5_S	Xisha islands maritime space, South China Sea	[37]
5*α*,8*α*-Epidioxy-cholest-6-en-3β-ol (**100**)	CH_2_Cl_2_ fraction of CH_2_Cl_2_/MeOH extract	416	C_27_H_44_O_3_	Xisha islands maritime space, South China Sea	[37]
Kauamide (**101**)	*n*-Hexane fraction of MeOH extract	357	C_19_H_33_ClNO_3_	Sheraton Caverns, Kauai, Hawaii	[25]

* Compound isolated from sponge’ s sample obtained from two different locations in the same study.

### 2.1. Sesquiterpenes

#### 2.1.1. Sesquiterpenic Quinones/Hydroquinones

Sesquiterpenic quinones/hydroquinones are a class of natural marine metabolites that are mainly reported from order Dictyoceratida sponges, including various genera such as *Dysidea*, *Fenestraspongia*, *Hyrtios*, *Dactylospongia*, *Petrosaspongia*, *Spongia*, and *Hippospongia* [27,32]. They can be either with the drimane or rearranged drimane (clerodane-decalin or 4,9-friedodrimane) skeleton, having four stereo-genic centers. The quinone/hydroquinone moiety could be mono, di, tri, tetra, or penta-substituted. These substituents can be hydroxy, methoxy, methyl ester, or amino groups. These metabolites possess variable structures based on the configuration at C-5 (cis- or trans-clerodane skeleton), C-9, C-8 and/or the position of the double bond. These metabolites have drawn remarkable interest due to their diverse structures and bioactivities.

Cancer represents one of the main reasons for death that has the second-highest incidence of mortality after cardiovascular diseases [38]. Chemotherapeutic treatment is the most common strategy for cancer treatment. However, the chemotherapeutic agents influence not only tumor cells but also normal cells resulting in hazardous side effects [39]. *D. elegans* reported sesquiterpenes have been evaluated for their anticancer potential towards various cancer cell lines. Further, some studies reported the structure–activity relationship and synthesis of some analogs have been discussed as shown here.

**Figure 1:**
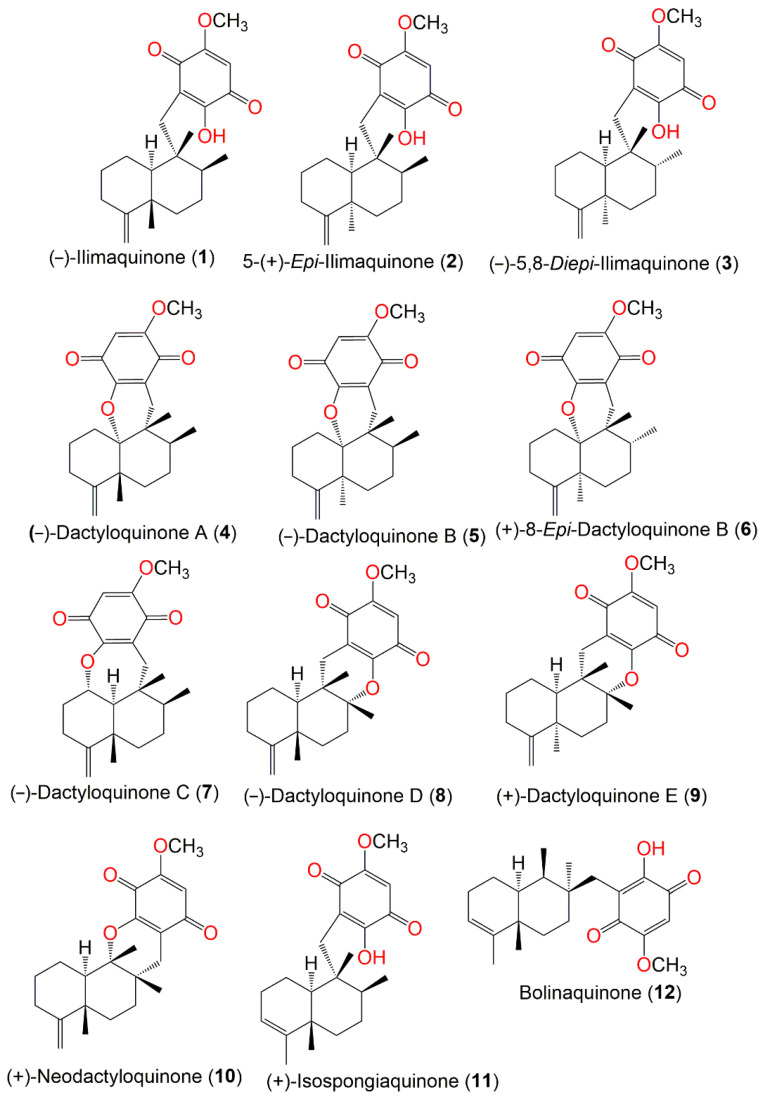
Structures of compounds **1**–**12**.

In 2017, Boufridi et al. assayed cytotoxic and apoptotic potential of (−)-ilimaquinone (**1**) and 5-(+)*-epi*-ilimaquinone (**2**) using Cell Titer-Glo luminescent cell viability assay towards HeLa (cervical adenocarcinoma), PC3 (prostate adenocarcinoma), MES-SA (uterine sarcoma), and MESSA/D×5 (multidrug-resistant uterine sarcoma) (Figure 1). These metabolites **1** and **2** possessed more potent growth inhibitory capacities against MES-SA and MESSA/D×5 (IC_50_ values of 2.44 and 2.56 µM for MES-SA and 10.48 and 12.54 µM, respectively, for MESSA/D×5). The incubation of MES-SA and MES-SA/D×5 cells with 5 and 50 µM of both **1** and **2** resulted in significant elevated caspases proteolytic activity in both cell lines, revealing the apoptotic capacity of both compounds. It is noteworthy that ilimaquinone had more potent cytotoxic and apoptotic potential than its C-5 epimer [40].

**Figure 2:**
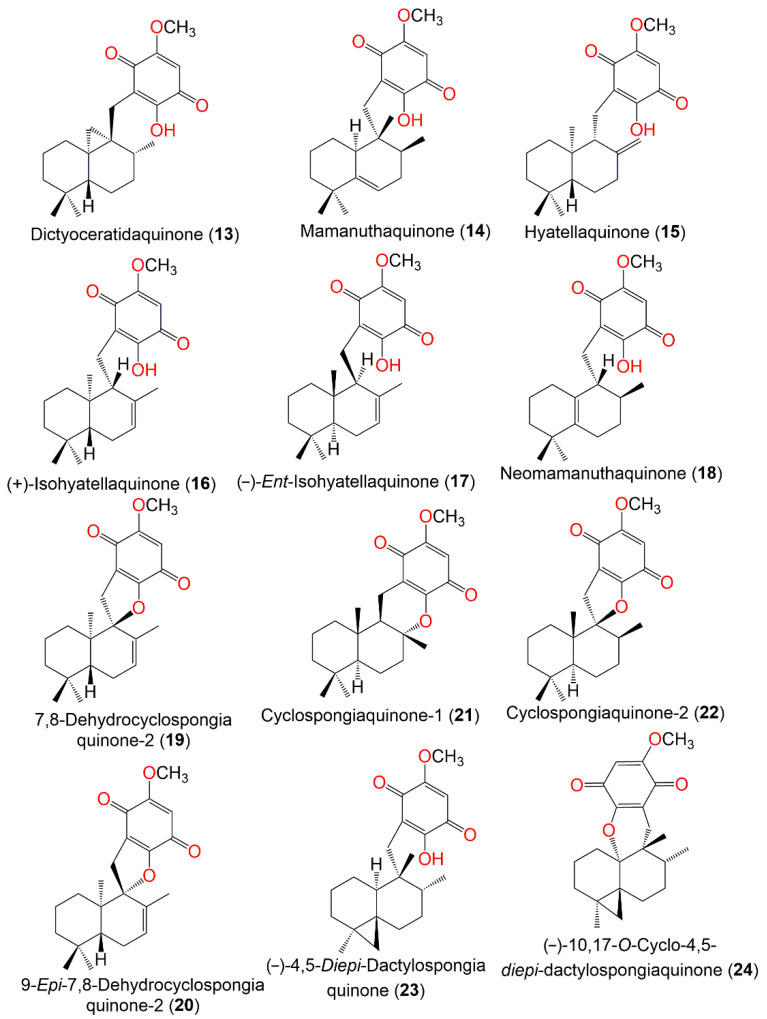
Structures of compounds **13**–**24**.

The new sesquiterpene quinones; (+)-isohyatellaquinone (**16**), (−)-*ent*-isohyatellaquinone (**17**), 7,8-dehydrocyclospongiaquinone-2 (**19**), and 9-*epi*-7,8-dehydrocyclospongiaquinone-2 (**20**), in addition to the known sesquiterpenes; **1**, **13**, **14**, and **15** were separated and purified from CH_2_Cl_2_/MeOH extract using AgNO_3_ (silver nitrate) flash chromatography (FC) and AgNO_3_-impregnated TLC (Figure 2). AgNO_3_ FC is an appropriate technique for the separation of metabolites with a difference in the alkene substitution pattern. Their relative configuration was assigned based on NOESY and optical rotation analyses. These compounds were identified by different spectroscopic techniques. Compound **13** featured an uncommon cyclopropyl moiety and trans-C-10 and C-5 ring junction, whereas **16** having drimane moiety linked to OH-substituted quinone, was structurally similar to **15** with an opposite optical rotation sign. On the other side, **19** is a dehydro-derivative of cyclospongiaquinone-2 (**22**), having a C-9-spiro center, while **20** differed from **19** in C-9-stereochemistry [21]. In cytotoxicity test, **1**, **14**, and **15** had potent potential (IC_50_ ranging from 1.50 to 4.45 µg/mL), compared to doxorubicin (IC_50_ 0.29 µg/mL) versus the BC cell line whereas **16** and **20** demonstrated moderate capacity (IC_50_ 6.69 and 7.38 µg/mL, respectively) in the MTT method. Only **1** (IC_50_ 3.37 µg/mL) possessed a strong potential versus NCI-H187 cell line in comparison to doxorubicin (IC_50_ 0.06 µg/mL) (Table 3).

A structure–activity relationship study revealed that 5,6-endocyclic double bond (e.g., mamanuthaquinone **14**), as well as exocyclic double bond (e.g., (−)-ilimaquinone **1** and hyatellaquinone **15**) contributed to a high potential towards the BC cell, while the exocyclic double bond (e.g., (−)-ilimaquinone **1**) could explain the capacity versus NCI-H187 cell line [21]. 

**Figure 3:**
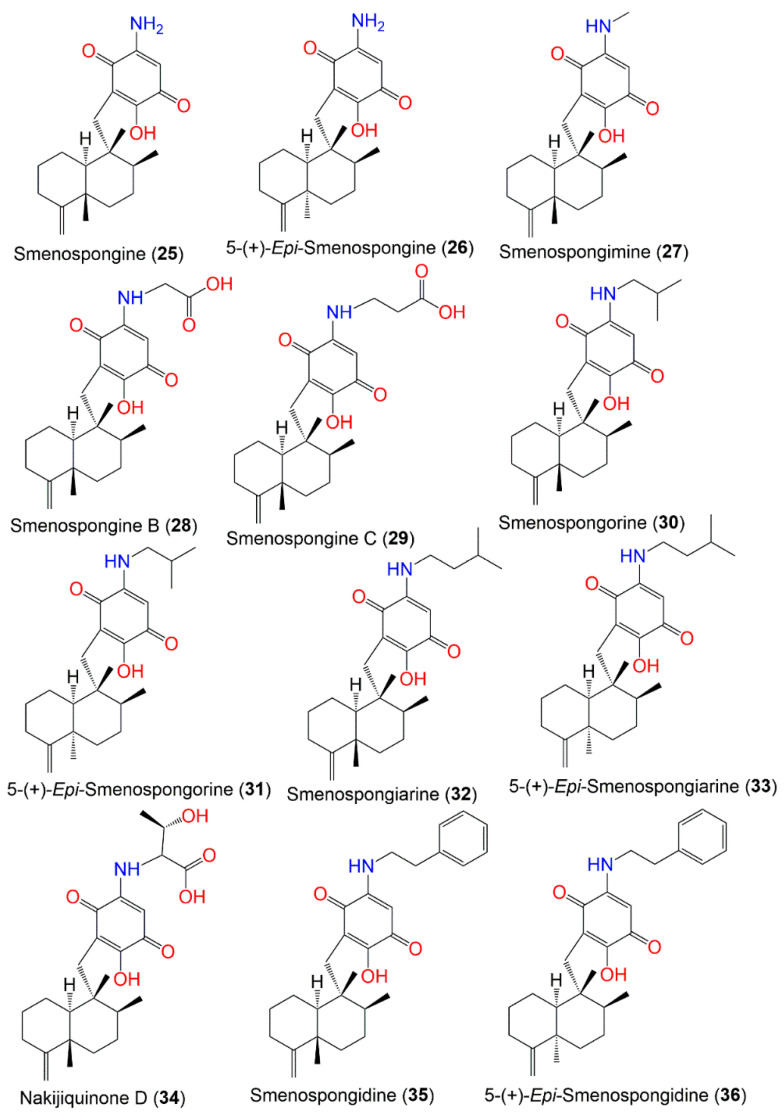
Structures of compounds **25**–**36**.

A new sesquiterpene benzoxazole; nakijinol B (**65**) and two new sesquiterpene quinones; smenospongines B (**28**) and C (**29**) along with (−)-ilimaquinone (**1**) and (−)-dactyloquinone B (**5**) were purified from methanol extract using RP-18 CC and HPLC. Their structures were elucidated based on spectroscopic analyses (Figure 3). Nakijinol B (**65**) had a *trans*-4,9-friedodrimane skeleton linked to benzoxazole moiety, while smenospongines B (**28**) and C (**29**) possessed 2-amino-acetic acid and 3-amino-propionic acid moiety at C-20, respectively. Their cytotoxic potential versus a panel of human tumor cell lines; SF-268 (central nervous system-glioblastoma cells), MCF-7 (breast-pleural effusion adenocarcinoma cells), H460 (lung-large cell carcinoma cells), HT-29 (colon-recto-sigmoid colon adenocarcinoma cells), and CHO-K1 (normal mammalian cell line) in the sulforhodamine B (SRB) assay were assessed. These metabolites demonstrated cytotoxic potential (GI_50_ 1.8–46 µM) and seemed to lack selectivity for tumor versus normal cell lines. Compound **1** was the most cytotoxic (GI_50_ values ranging from 1.8 to 5.4 µM). The additional methylene in the *N*-substituted side chain (e.g., **29**) reduced observed activity by two-fold than **28** [22].

**Figure 4:**
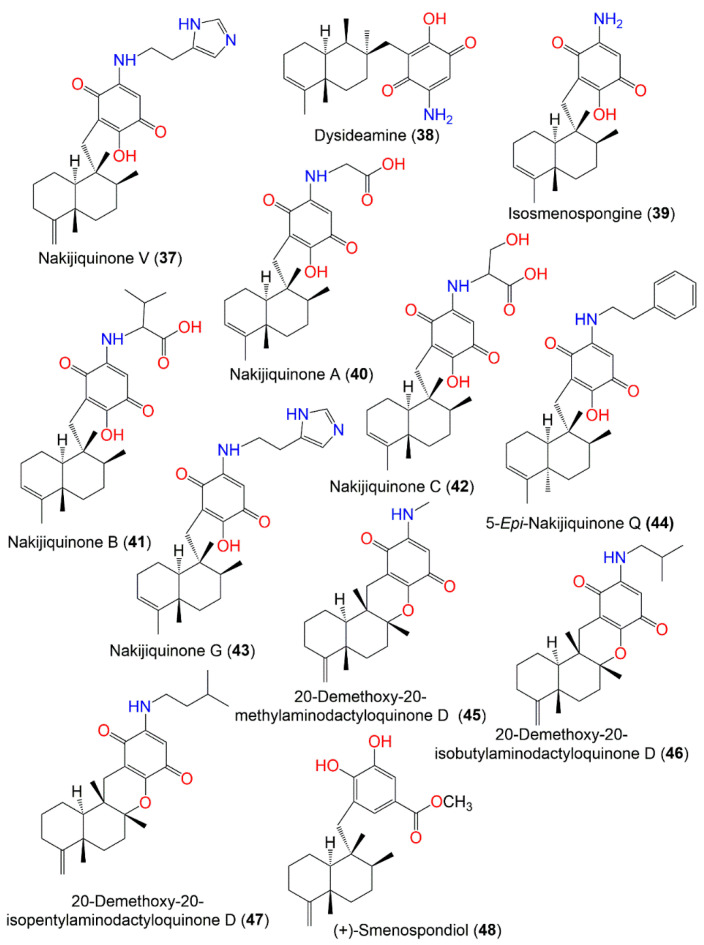
Structures of compounds **37**–**48**.

In 2019, Neupane et al. purified **1**, **2**, **25**, **30**, **32**, **35**, **49**, **51**, and **52** using flash SiO_2_ CC and RP-HPLC. Compounds **1** and **25** moderately prohibited BACE1 (β-secretase 1), an enzyme involved in Alzheimer’s disease pathogenesis, however, the other metabolites had no or weak activity (Figure 4). On the other side, **1**, **2**, **25**, **30**, **32**, **35**, **49**, **51**, and **52** revealed cytotoxic potential (CC_50_ from 2.4 to 19.4 µM) versus U251MG cells with, with **25** and **49** having the most potent influences (CC_50_s 2.4 and 2.8 µM, respectively) [26]. Further, **1**, **2, 30**, **32**, **35**, **49**, and **51** were significantly active versus Panc-1 (human pancreatic cancer) cells (CC_50_s ranged from 12.6 to 22.6 µM) [26]. 

**Figure 5:**
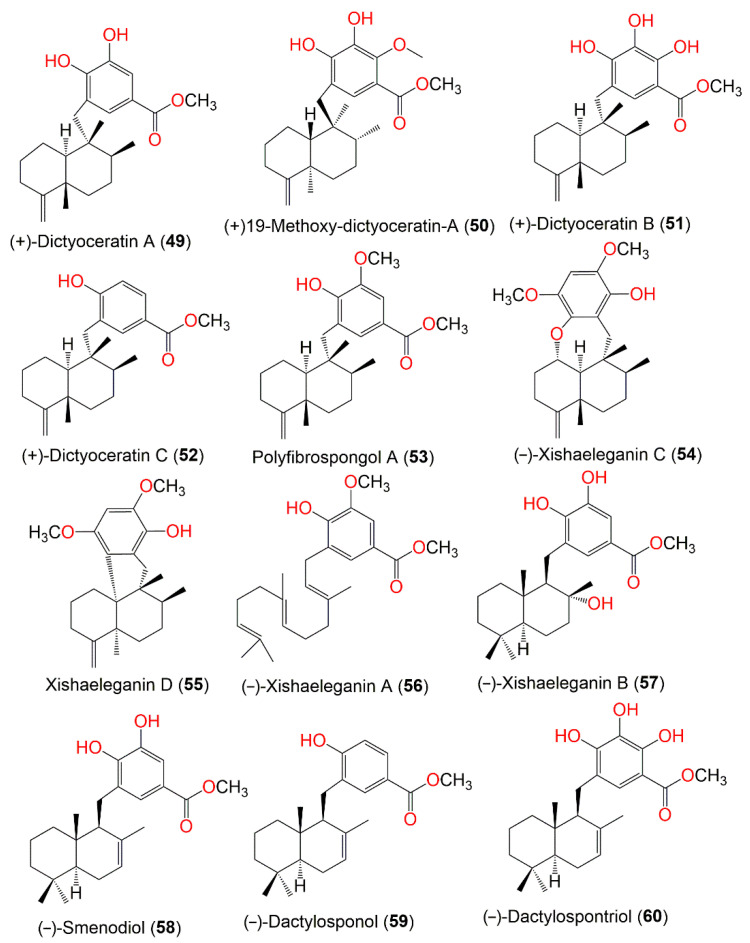
Structures of compounds **49**–**60**.

Using SiO_2_, RP-18, and HPLC, three new sesquiterpenes; **46**, **47**, and **50**, along with **27**, **30, 32**, **45**, and **52** were isolated. Their structures were verified using spectroscopic, ECD (electronic-circular-dichroism), and X-ray analyses (Figure 5). Compounds **46** and **47** possessed an *O*-bridge among C-17 and C-8 and 5S,8R,9S,10R absolute configuration, as well as isobutylamino and isopentylamino groups, respectively, at C-20; whilst **50** was a hydroquinone sesquiterpene, having 5R,8R,9S,10R configuration based on X-ray and CD analyses. 

Their cytotoxic potential was assessed versus SW1990 (human pancreatic cancer), DU145 (human prostate cancer), Huh7 (human liver cancer), and Panc-1 cell lines in the CCK-8 (cell counting kit-8) assay. Compounds **27**, **30**, **32**, and **50** showed activities versus all cell lines (IC_50_s from 2.33 to 37.85 µM), whereas **45**–**47** showed no cytotoxicity. It was found that C-8 and C-17 cyclization via *O*-atom resulted in the loss of inhibitory activity as in **45**–**47** (IC_50_ > 50 µM), in comparison to **27**, **30**, and **32** (IC_50_ 2.33–9.20 µM) [32].

Rodriguez et al. stated that the quinone ring was substantial for the in vitro cytotoxic potential versus the solid tumors, as displayed by the 5-(+)-epi-ilimaquinone (**2**) and 5-(+)-epi-smenospongiarine (33) potency and dactylospongenones A–D (**81**–**84)** inactivity [16]. 

Differentiation induction therapy is one of the alternative therapeutic methods that is based on the differentiation of tumor cells to normal cells using a differentiation inducer, ATRA (all-trans-retinoic acid) [41]. However, different types of leukemia were found to be unresponsive to ATRA, such as CML (human chronic myelogenous leukemia). So, several exploratory research was carried out to discover new differentiation inducers from marine organisms. CML is a hematopoietic stem cell cancer produced by the Bcr-Abl tyrosine kinase that is resulted from Philadelphia chromosome (Ph) translocation [42]. Allogeneic BMT (bone marrow transplant) is the only common curative therapy for CML. However, the treatment-associated toxicity is dangerous with about 30% reported mortality [43]. Aoki et al. stated that smenospongine (**25**) (Conc. 3–15 µM) induced K562 CML cells differentiation into erythroblasts alongside with cell cycle arrest at the G1 phase and increased expression of p21 protein, which had an important role in differentiation. Further, it prohibited the phosphorylation of Crkl, which is a substrate of Bcr-Abl tyrosine kinase [31]. Smenospongine **25** an aminoquinone sesquiterpene was firstly reported in 1987 as an antimicrobial and cytotoxic metabolite from *Smenospongia* sp. [44].

Further, in 2008, Kong et al. investigated the influence of **25** on the cell cycles of various cells, including HL60 (human acute promyelocytic leukemia) and U937 (human histiocytic lymphoma) cells, as well as the mechanism of K562 cells G1-phase arrest. It was found to induce dose-dependent apoptosis in U937 and HL60 cells and G1 arrest in K562 cells. In K562 cells, it boosted p21 expression and suppressed Rb phosphorylation, revealing the remarkable function of the p21-Rb pathway in G1 arrest. In addition, it could enhance p21 expression through another mechanism than p21 promoter transactivation [43]. Further, its effect versus a panel of 39 solid cancer cell lines was assessed in the SRB and WST-8 (water-soluble tetrazolium salt-8) assays. Smenospongine (**25**) suppressed the growth of these cells in vitro (mean Log GI_50_ -5.55). Additionally, it prohibited migration, proliferation, and HUVEC (human umbilical vein endothelial cells) tube formation. Hence, it demonstrated antitumor potential versus solid tumors through direct growth inhibition of the tumor cells and anti-angiogenic effectiveness on endothelial cells, indicating its potential as a lead compound for discovering a prominent anticancer [45]. 

In another study by Aoki et al., a new aminoquinone sesquiterpene, 5-(+)-*Epi*-Smenospongorine (**31**) and known quinone/hydroquinone sesquiterpenes; **1**, **2**, **25**, **26**, **30**, **35**, **36**, **48**, and **52** were separated. Compound **31** was presumed to be a hybrid of **30** and **36** and identified a C-5 epimer **30** as confirmed by NOESY (nuclear Overhauser effect spectroscopy). These metabolites were assessed for differentiation-producing potential by induction of hemoglobin production in K562 cells, where the hemoglobin pseudo-peroxidase effect was estimated colorimetrically using diaminofluorene. It was found that **25**, **26**, **30**, **31**, **35**, and **36** possessed similar K562 cells differentiation-inducing capacity into erythroblasts and were more powerful than aphidicolin; while **48** and **52** had no activity and **2** and **1** had only activity at a higher concentration than those of **25**, **26**, **30**, **31**, **35**, and **36** [19]. Structure–activity relationship studies revealed that quinone moiety and amino group were crucial for the activity, whereas the substituents at the amino group and C-5 configuration were not essential [20].

A tumor environment’s hypoxic condition is now known as an essential factor for angiogenesis, tumor growth, and metastasis; additionally, at this condition the tumor cells become resistant to irradiation and chemotherapy [23,24]. Thus, the metabolites that selectively prohibit tumor cells growth in the hypoxic environment are expected to be a promising new lead for anticancer agents.

Hypoxia-inducible factor-1 (HIF-1) is a hetero-dimeric transcription factor that comprises an O_2_-regulated α-subunit and a constitutively expressed β-subunit. Hypoxia prohibited the HIF-1α subunit O_2_-dependent hydroxylation, leading to degradation by the proteasome, dimerization of accumulated HIF-1α with HIF-1β, and activation of target genes transcription. HIF-1 activation enhances cancer progression and/or oncogenesis. Furthermore, HIF-1 inhibition causes a decrease in VEGF (vascular endothelial growth factor) expression [24]. As such, HIF-1 has been drawn much interest as a target for chemotherapeutic drugs.

The new metabolites: (−)-5,8-diepi-ilimaquinone (**3**), (+)-8-epi-dactyloquinone B (**6**), and 4,5-diepi-dactylospongiaquinone (**23**), along with **1**, **21**, **22, 24**, **61**, and **63** were purified from CH_2_Cl_2_ fraction of H_2_O extract SiO_2_ and RP-18 CC and characterized by intensive spectroscopic techniques [23]. Compound **3** was a new stereoisomer of **1** and **2**, which was identified as a C-8 epimer of **2**. On the other side, **23** had C4-C5 cyclopropyl ring instead of the C5-C11 exomethylene in **3** and was assigned as dactylospongiaquinone cyclopropyl inverted analog. Additionally, **6**, a C-8 epimer of (−)- dactyloquinone B (**5)** possessed a dihydro-pyran ring that was formed by a C10-O-C17 connection between 4,9-friedodrimane and dialkoxy-1,4-benzoquinone. The activation capacity of these metabolites towards HIF-1 was estimated utilizing colorimetric BCA protein Assay Kits, as well as their cytotoxicity versus MDA-MB-231 and T47D cells in the SRB assay was evaluated [23]. Compounds **1**, **3**, and **23** with a 2-hydroxy-5-methoxy-1,4-benzoquinone moiety activated HIF-1 at concentrations of 10 and 30 μM. They possessed a high level of HIF induction (930%, 830%, and 1000%, respectively) at 10 μM. However, other compounds had no significant activity, suggesting the 2-hydroxy-1,4-benzoquinone moiety was essential for HIF-1 activation. Further, **1**, **3**, and **23** (10 μM, 16 h) raised both cellular and secreted VEGF proteins levels in T47D cells similar to 1,10-phenanthroline. VEGF is a HIF-1 target gene that boots angiogenesis. On the other hand, all metabolites except **63** prohibited T47D cell proliferation more effectively than MDAMB-231 cells, suggesting that the pharmacophores accountable for cell proliferation inhibition and HIF activation were not identical. It was found that the substituted 1,4-benzoquinone’s OH group was substantial for the HIF-1 activating potential (e.g., **1**, **3**, and **23**), whereas its change by ring formation or exchange with a phenol completely abolished the activity [23].

A sesquiterpene phenol, (+)-dictyoceratin C (**52**) (Conc. 1.0⁠–⁠10 μM) selectively prohibited the DU145 cells proliferation under hypoxic conditions through suppression of HIF-1 accumulation under hypoxic conditions. A structure–activity relationship study was reported utilizing previously reported **1**, **12**, **25**, **26**, **30**, **31**, **36**, **38**, and **48**. (+)-Smenospondiol (**48**) also demonstrated a similar hypoxia-specific growth inhibition capacity versus DU145 cells as **52**, revealing para-hydroxy benzoyl ester moiety was substantial for activity, while the chiral decalin skeleton had no role for activity. On the other side, compounds **1**, **12**, **25**, **26**, **30**, **31**, **36**, and **38** containing hydroxyquinone moiety did not demonstrate hypoxia-selective growth inhibition [24]. 

Additionally, in 2015 Sumii et al. reported the selective prohibition of DU145 proliferation by **49** and **52** under hypoxic conditions and their in vivo antitumor effects in subcutaneously inoculated mice with sarcoma S180 cells with no observed acute toxicities during the study period for these compounds [46]. It was implied that methyl ester, exo-olefinic bond, 8-methyl, and OH group were crucial for the hypoxia selective growth inhibition activities of **49** and **52** [45]. Collectively, not only the *para*-hydroxy-benzoyl moiety but also the decalin skeleton with 8-methyl and 4-*exo*-cyclic olefinic bond were significant for hypoxia-targeted growth inhibition of **49** and **52** [46,47].

Goclik et al. purified and characterized a new sesquiterpene related to hydroquinones; pelorol (64) and drimane sesquiterpene, 1 from the CH_2_Cl_2_ soluble fraction using extensive SiO_2_ CC and spectroscopic analyses. Compound 64 is a sesquiterpene hydroquinone derivative, having tetracyclic structure with a pentacyclic ring. They had weak anti-trypanosomal and antimalarial potential towards *Trypanosoma brucei* (IC_50_ 17.4 and 7.7 µg/mL, respectively) and *Plasmodium falciparum* clone K1 and clone NF54 (IC_50_ 786.0 and 1911.0 µg/mL for 64, and 1743.0 and 949.0 for 1, respectively), respectively, in comparison to melarsoprol (IC_50_ 0.0026 µg/mL for *T. brucei*) and chloroquine (IC_50_ 91.0 and 4.6 µg/mL for clone K1 and NF54, respectively) in the semiautomated microdilution method [18]. Additionally, **1** possessed TK (tyrosine kinase) inhibitory effectiveness (87% at Conc. 1.0 µg/µL) [18]. 

Ebada et al. separated two new drimane sesquiterpenes; dactylospongenones G (**85**) and dactylospongenone H (**86**) in addition to, **2**, **11**, **26**, **29**, **34**, **36**, **39**–**44**, and **64** from the *n*-hexane soluble fraction by SiO_2_ CC and HPLC that were unambiguously characterized by NMR spectroscopy and HRESIMS. Dactylospongenones G (**85**) and dactylospongenone H (**86**) were isolated as an inseparable mixture, possessing cis 4,9-friedodrim-4(11)-ene and trans-4,9-friedodrim-3(4)-ene skeleton, respectively, with cyclopentadienone moiety. Among these metabolites, **36**, **11**, **2**, **39**, **40**, and **43** exhibited potent cytotoxic potential (IC_50_ ranging from 1.3 and 6.48 µM) in comparison to kahalalide F (IC_50_ 4.30 µM) versus L5178Y (mouse lymphoma) cell line. They were also assessed for antimicrobial capacity versus *S. aureus* ATCC-25923, *S. aureus* ATCC-700699, *E. faecalis* ATCC-29212, *E. faecalis* ATCC-51299, *E. faecalis* ATCC- 35667, and *E. faecalis* ATCC-700221 utilizing a broth microdilution method. Pelorol (**64**) demonstrated moderate to potent antibacterial capacity versus the tested microorganisms (MICs 3.125 to 25 µM) with high effect towards *S. aureus* (MIC 3.125 µM). None of them had antitubercular capacity versus *Mycobacterium tuberculosis* [28].

Nakijiquinone V (**37**), a new aminoquinone sesquiterpenoid, in addition to **1**, **25**, and **52** were elucidated using NMR and LC-HRESIMS techniques. Nakijiquinone V (**37**) had Δ^4,11^ friedodrimane quinone skeleton and an imidazole ring connected to the quinone moiety via an amino ethylene group. These metabolites were assessed for antibacterial capacity versus *M. luteus* ATCC-4698, *B. megaterium* DSM-32, and *E. coli* K12. Compounds **1**, **25**, and **52** had moderate to weak effectiveness versus *B. megaterium* DSM32 (MICs 32, 32, and 64 µg/mL, respectively). Further, **1** and **25** (MIC of 32 µg/mL) prohibited *M. luteus* ATCC-4698 [25]. 

New sesquiterpene hydroquinones; (−)-xishaeleganin A (**56**), B (**57**), C (**54**), and D (**55**), along with known related metabolites **1**, **2**, **4**, **5**, **7**–**9, 49**, **51**, **52**, and **53** were separated from Et_2_O fraction of acetone extract using SiO_2_, Sephadex LH-20, and RP-HPLC. Compound **56**, hydroquinone sesquiterpene with farnesyl moiety was related to 4-hydroxy-3-methoxy-5-((2E,6E)-3,7,11-trimethyldodeca-2,6,10-trien-1-yl)benzoic acid reported from *Aspergillus flavipes* [48]. Additionally, **57** is a drimane sesquiterpene similar to *ent*-yahazunol reported from *Dysidea* genus sponge [49], however, it showed 1,2,3,5-tetrasubstituted hydroquinone, instead of a 1,2,4-tri-substituted hydroquinone. Compounds **54** and **55** are 4,9-friedodrimane sesquiterpene hydroquinones in which the sesquiterpene skeleton connected to hydroquinone through C1–C17 ether linkage and direct C10–C17 carbon linkage, respectively, to produce a seven-membered ring (in **54**) and five-membered ring (in **55**). These metabolites were tested for antibacterial potential versus *S. aureus* USA300-LAC, *S. pyogenes* ATCC-12344, and *E. faecium* Efm-HS0649 in the broth microdilution method. Compounds **1**, **2**, **49**, and **57** had marked antibacterial capacity versus *S. aureus* (MICs 5.6, 5.6, 2.9, and 1.5 µg/mL, respectively) in comparison to vancomycin (MIC 1.0 µg/mL). As for *S. pyogenes*, compounds **1**, **2**, **7**, **49**, **51**, **54**, and **57** demonstrated significant antibacterial effectiveness (MICs ranged from 1.5 to 5.6 µg/mL). In addition, compounds **7**, **49**, **51**, **54**, and **57** displayed significant potential versus *E. faecium* (MICs 1.4–5.6 µg/mL). These results suggested that the sesquiterpene quinones/hydroquinones as **57** and **49** possessed the possibility to be the new lead metabolites of antibiotics [27].

#### 2.1.2. Sesquiterpenic Quinone/Hydroquinone Dimers

**Figure 6:**
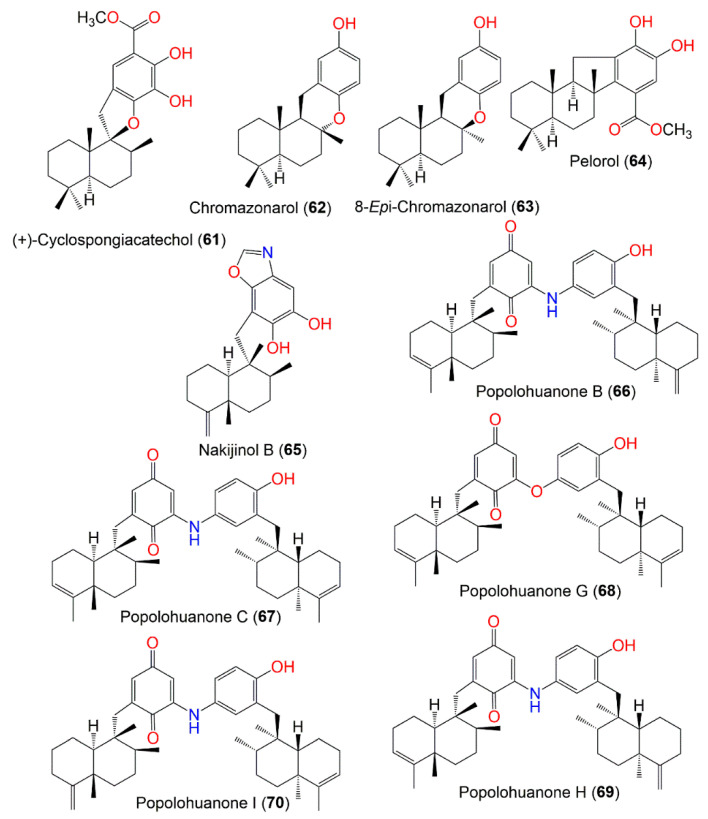
Structures of compounds **61**–**70**.

Li et al. reported the characterization of popolohuanones B (**66**), C (**67**), G (**68**), H (**69**), and I (**70**) that are dimeric sesquiterpenes, having linked quinone and hydroquinone moieties through either amine or ether bridge (C_19_-O-C20’ as in **66**, C19-N-C20’ as in **67**, or C18-N-C20’ as in **68**, **69**, and **70**). In the CCK-8 cytotoxicity assay, these metabolites had no cytotoxic potential versus HT-29, PC-9, A375, HepG2, and MCF-7. On the other hand, only **69** displayed potent IL-6 production inhibitory influence that was induced by LPS in the THP-1 cells with (%inhibition 73.1%, Conc. 10 µM). It was assumed that the C_19_–NH–C_20_’ amine bridge among the hydroquinone and quinone moiety could be the active moiety for popolohuanones [34] (Figure 6).

#### 2.1.3. Sesquiterpene Tetronic Acids

**Figure 7:**
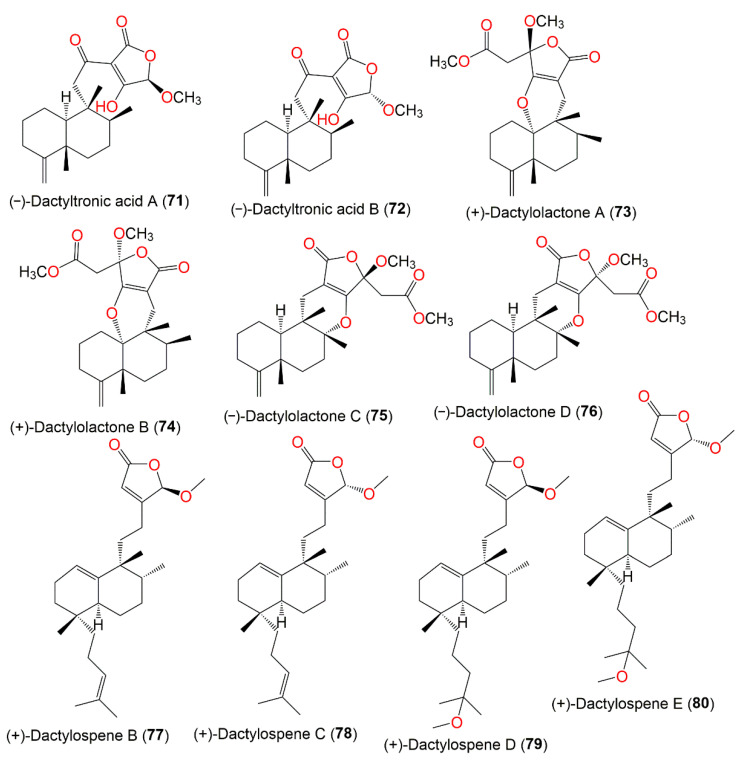
Structures of compounds **71**–**80**.

Sesquiterpene tetronic acids are a rare class of sesquiterpenes with 4-hydroxy-[5H] furan-2-one moiety linked to the sesquiterpene skeleton. (−)-Dactyltronic acids (A/B **71/72**) were separated as a mixture of inseparable isomers from the EtOAc by SiO_2_ and Sephadex CC, as well as HPLC. Dactyltronic acids A/B (**71**/**72**) had pronounced antibacterial potential towards *V. parahemolyticus* (MIC 3.45 µM) relative to ciprofloxacin (MIC 1.25 µM), whereas compounds **52** and **53** were weakly active [33] (Figure 7).

### 2.2. Sesterterpenes

Chemical investigation of *D. elegans* afforded new γ-oxygenated utanolide sesterterpene derivatives; dactylospenes A (**95**) and B-E (**77**–**80**), in addition to structurally related compounds; furospinosulin B (**93**) and (−)-luffariellolide (**94**) that were characterized based on spectroscopic and ECD analyses. Only compounds **78**, **94**, and **95** (IC_50_s 2.11–13.35 µM) possessed moderate cytotoxic potential versus Huh7, DU145, SW1990, and PANC-1 in the CCK-8 assay. These results indicated that *R*-γ-methoxy utanolide unit influenced positively the activity [35]. 

Arai et al. found that the furanosesterterpene; furospinosulin-1 (**92**) (Conc. 1⁠–⁠100 µM) demonstrated an in vitro selective antiproliferative potential versus DU145 (human prostate cancer cells) under hypoxic conditions [50]. Additionally, it had antitumor potential without adverse influences upon administration (Conc. 10–50 mg/kg, orally) in a sarcoma S180-inoculated mouse model. Further mechanistic studies indicated that **92** repressed *IGF-2* (insulin-like growth factor-2) gene transcription that is selectively produced under hypoxia through the prohibition of the nuclear proteins binding to the Sp1 consensus sequence in the *IGF-2* promoter region, while it did not prohibit HIF-1α production (hypoxia-inducible factor-1α) [49,50]. It could also prohibit IGF-IR signaling (insulin growth factor 1 receptor) through IGF-2 transcription suppression [51]. Accordingly, furospinosulin-1 may be a potential lead for anticancer agents, which target hypoxia-acclimatized cancer cells [49]. Furospinosulin-1 (**92**) also had inhibitory potential versus HCT-116 (human colon cancer, IC_50_ 155 µM) and Cdc25A (IC_50_ 2.5 µM) [52,53].

### 2.3. Diterpenes

Dolabellane diterpenes are diterpenoids with a dolabellane skeleton, consisting of an unusual *trans*-bicyclo [9.3.0]tetradecane nucleus. A rare diterpene, (+)-eleganstone A (**88**), having a 5/6/4/5-fused tetracyclic skeleton and a new dolabellane diterpene; (+)-(1R*,2E,4R*,8E,10S*,11S*,12R*)-10,18-diacetoxydolabella-2,8-dien-6-one (**90**), and formerly reported **89** and **91** were separated from CH_2_Cl_2_ fraction using RP-18 and HPLC and elucidated by spectroscopic and ECD analyses. Compounds **89** and **91** are a pair of Z/E isomers that were interconverted by light-induced isomerization. These metabolites had no cytotoxic effectiveness (IC_50_ > 50 µM) versus HCT-116, 22RV1 (human prostate carcinoma epithelial cell line), MCF-7 (human breast cancer), and K562 (human chronic leukemia) in the CCK-8 assay. On the other hand, only **89** demonstrated potent antibacterial capacities towards *E. coli*, *B. subtilis*, and *S. aureus* (MIC 32 µg/mL) in the broth dilution method [36].

### 2.4. Sterols and Pregnanes

**Figure 9:**
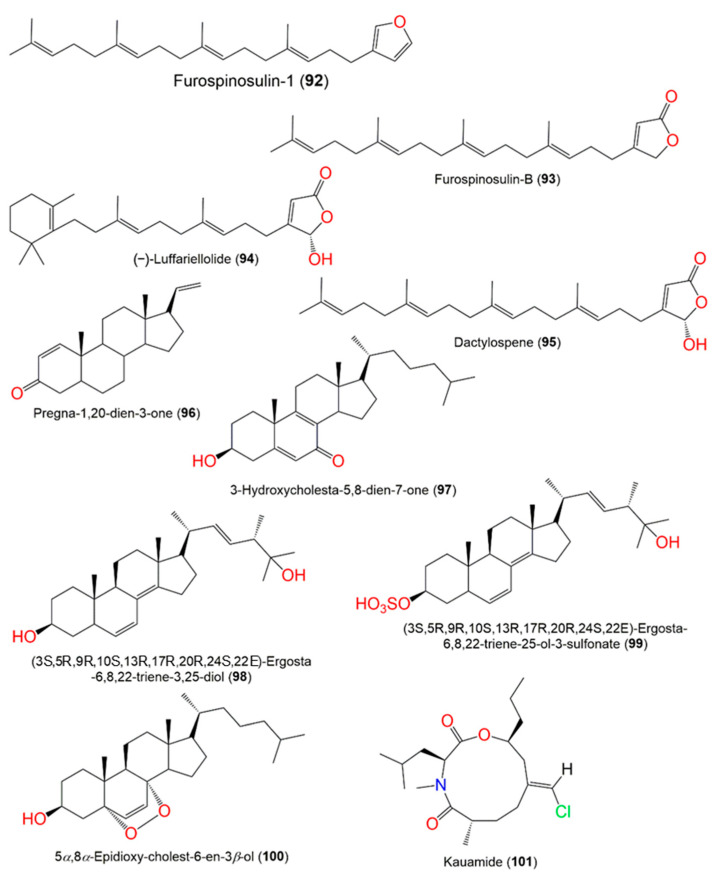
Structures of compounds **92–101**.

Pregna-1,20-dien-3-one (**96**) and 3-hydroxycholesta-5,8-dien-7-one (**97**) were purified from the EtOAc fraction. Compound **96** displayed antibacterial potential (MIC 4.19 µM) versus *B. cereus*, comparing to ciprofloxacin (MIC 1.25 µM), while **97** had a weak activity [33] (Figure 9).

The cytotoxicity assessment using CCK-4 assay of steroid derivatives; 98–100 versus HepG 2, HT-29, and MCF-7 revealed that **98** and **99** displayed notable cytotoxic capacities versus MCF-7 (IC_50_ 9.7 and 8.5 µM, respectively). However, they had weak to no activity towards the other cell lines [37].

### 2.5. Other Metabolites

Neupane et al. separated kauamide (**101**), a new chlorinated metabolite with a rare 11-membered heterocyclic skeleton. The structure of **101** was verified by spectroscopic analyses and its 3*S*, 6*S*, 11*S*, and L-leucine stereoconfigurations were established from GIAO (gauge-independent atomic orbital) NMR shielding tensors DFT (density functional theory) calculations, and Marfey’s analysis. It had no BACE1 inhibitory potential and cytotoxic activity against U251 and Panc-1 cell lines in the MTT assay [26].

## 3. Biosynthetic Pathways of *D. elegans* Metabolites

Several studies reported the biosynthetic pathways of the reported sesquiterpenes from this sponge. In this work, the reported postulated pathways were summarized. It was reported that the observed differences in stereochemistry among the marine sesquiterpene metabolites could be inferred from the precursor binding preferences within a single cyclase enzyme active site [54]. Additionally, this may be due to the existence of various synthase enzymes, whereas each individual enzyme can create a range of diverse metabolites, as well as presumably the potential to change the stereochemical outcomes, relying on the provided substrate nature. Thus, the possibility of enantiomeric metabolites should be considered that emphasize the significance of reporting [α]_D_ values for these terpenes in cases where they are utilized as a reference in the stereochemical determination [21,55].

**Scheme 1:**
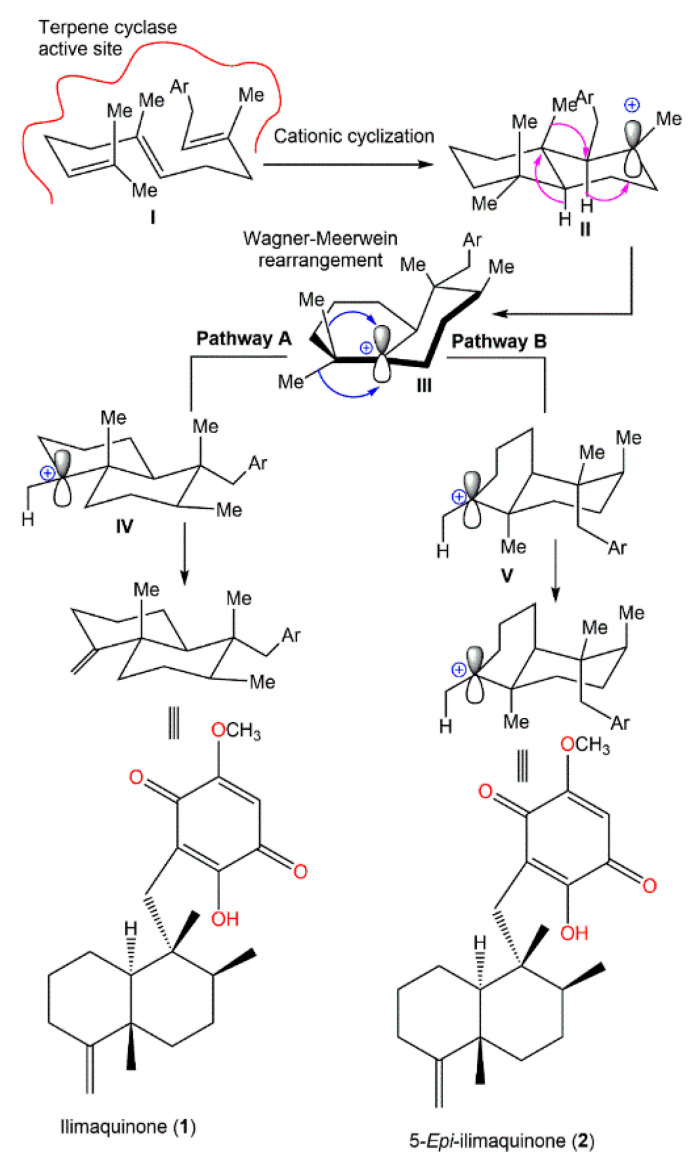
Postulated biosynthetic pathway for **1** and **2** [40].

Boufridi et al. hypothesized that the biosynthetic process of **1** and **2** started with the farnesylation of the aromatic ring, which is the quinone moiety’s precursor to obtain **I**. This involves the initial folding of **I** within the active site of a specific terpene cyclase (Scheme 1). Successively, two carbocationic intermediates **II** and **III** are resulted from peri-planar Wagner–Meerwein hydrogen and methyl shifts. Finally, **III** may undergo two pathways (A or B) for the formation of **1** and **2** through the loss of a proton from carbocations **IV** and **V**, respectively [40]. 

**Scheme 2:**
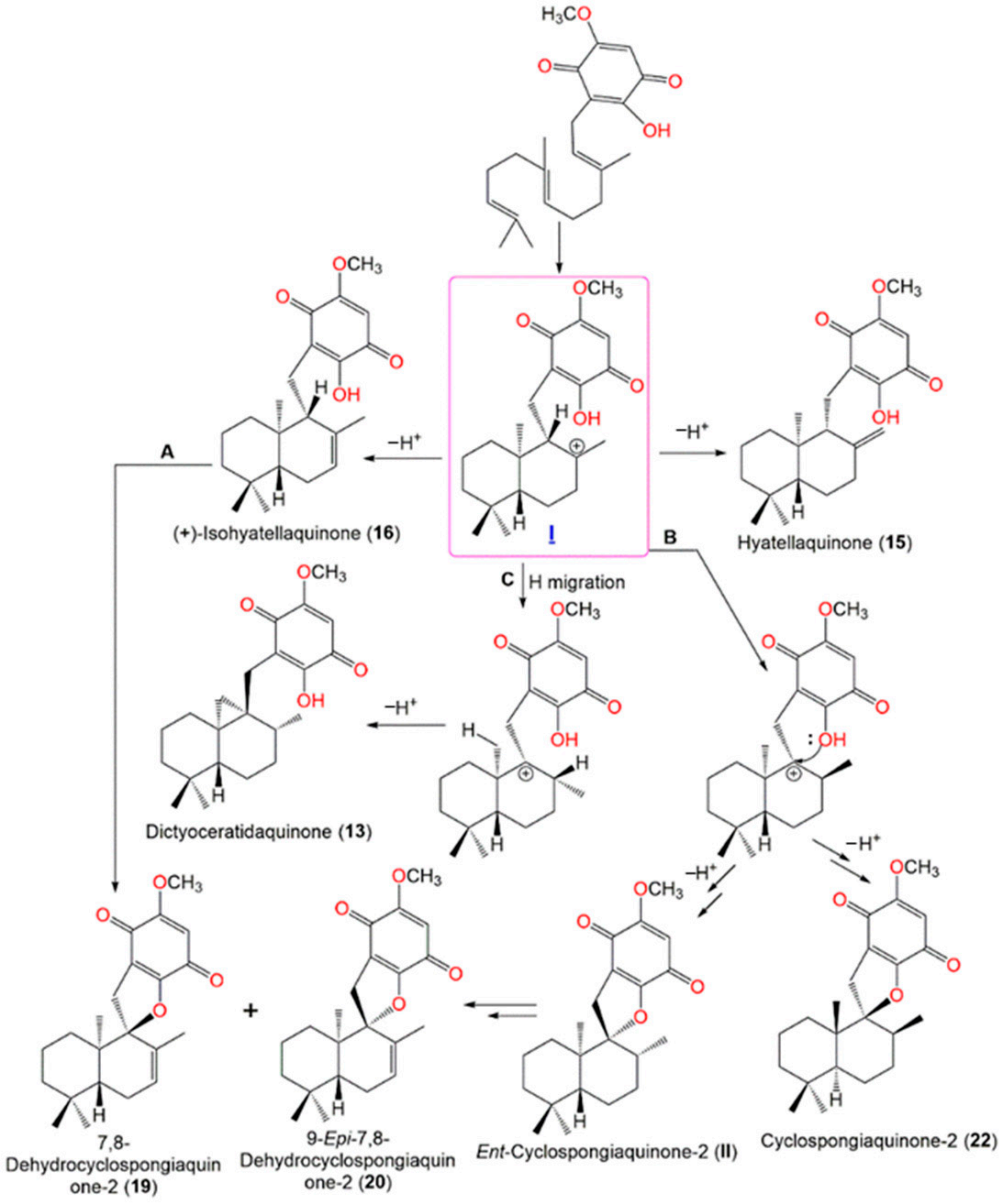
Postulated biosynthetic pathways for **13**, **15**, **16**, **19**, **20**, and **22** [21].

Yong et al. postulated that **13**, **15**, **16**, **19**, **20**, and **22** may be originated from the cationic intermediate (**I**) (Scheme 2). The introduction of double bond yields **16**. The cyclization of **16** (pathway A), or hydride migration with subsequent cyclization forming **22** and its related cyclic intermediate *ent*-cyclospongiaquinone-2 (**II**), then the formation of double bond yields **19** and **20** (pathway B). Finally, the hydride migration and loss of a proton from the cationic site adjacent methyl form **13** with a cyclopropyl ring (pathway C) [21].

**Scheme 3:**
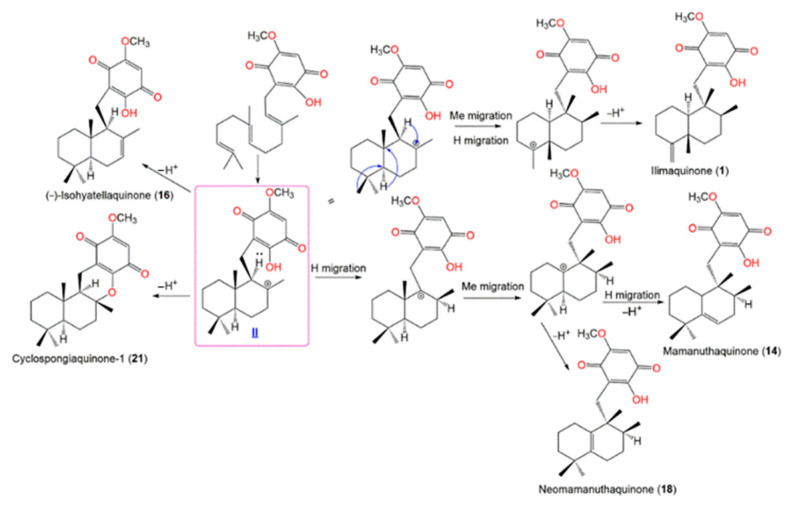
Postulated biosynthetic pathways for **1**, **14**, **16**, **18**, and **21** [21].

Scheme 3 shows that the farnesyl precursor (**I**) initial cyclization gives **II** (enantiomeric cation) that undergoes a loss of a proton to give **16** and **21**. On the other side, cation **II**’s hydride and methyl migrations yield compounds **1**, **14**, and **18** [21].

**Scheme 4:**
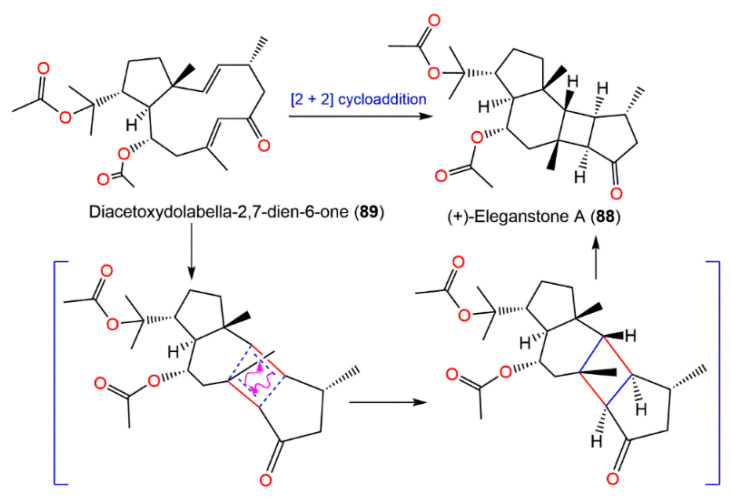
Proposed biosynthetic pathway for **88** [36].

Diacetoxydolabella-2,7-dien-6-one (**89**) was proposed to be the biogenetic precursor of **88**. (+)-Eleganstone A (**88**) may be originated from diacetoxydolabella-2,7-dien-6-one (**89**) through intramolecular [2 + 2] cycloaddition. The coupling of Δ^7,8^ and Δ^2,3^ in **89** via the endo-cycloaddition generates **88** (Scheme 4) [36,56].

## 4. Synthesis of *D. elegans* Metabolites

Some of the reported metabolites from this sponge possessed fascinating bioactivities, such as anticancer. Nevertheless, further biological investigation is limited due to the not enough isolated metabolites. Therefore, research interests have been directed towards synthesis and structural modification of these metabolites to improve the bioactivities and study structural/activity relations, which could help in drug development and discovery. Some of these studies have been highlighted here.

**Scheme 5:**
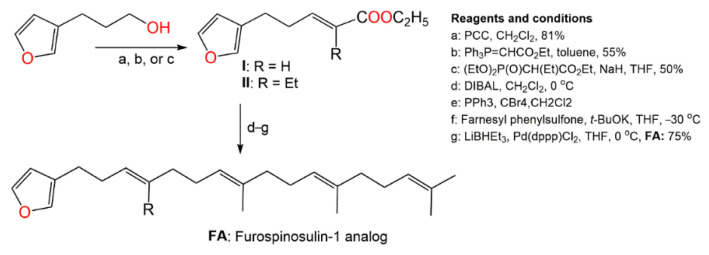
Synthesis of furospinosulin-1 (**92**) analogues [57].

Kotoku et al. synthesized several analogs of furospinosulin-1 (**92**) and assessed their selective hypoxia inhibitory potential (Scheme 5) [57]. 

It was found that only the analog (**FA**) with desmethyl near to the furan ring had an excellent hypoxia-specific growth inhibitory potential such as furospinosulin-1 (**92**) and displayed greater in vivo antitumor potential in oral administration (doses 1–10 µM) versus DU145 cells, as well as lower toxicity in normal conditions (dose 300 µM) [57]. Therefore, this analog might be better than furospinosulin-1 for drug candidates.

**Scheme 6:**
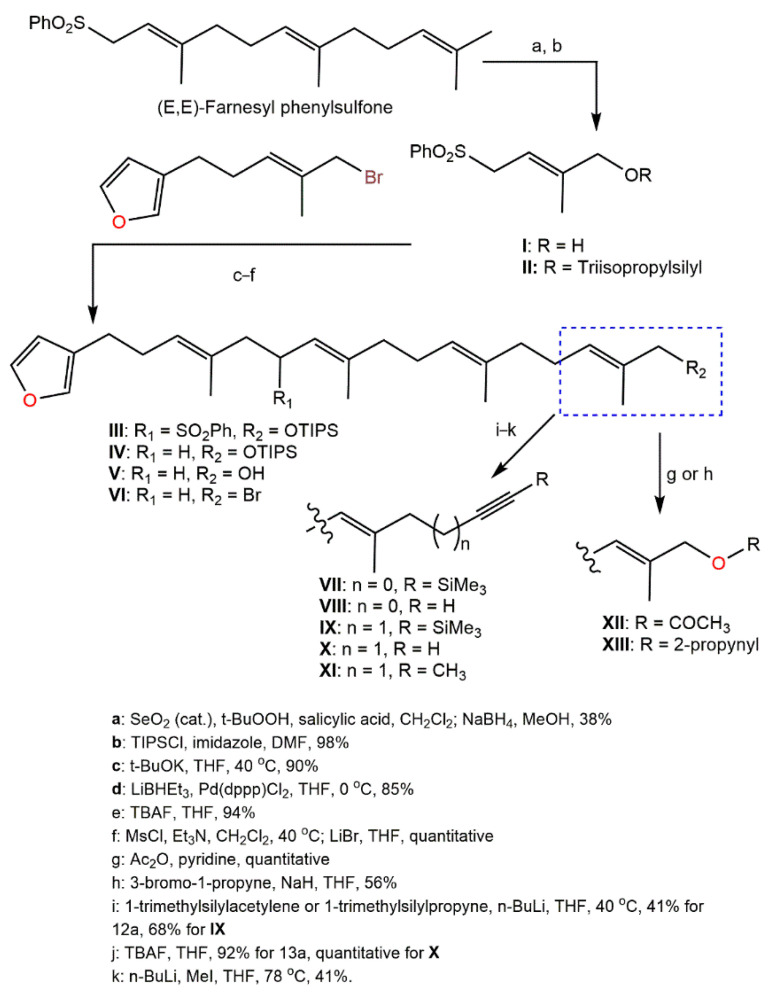
Synthesis of tail-modified analogues of furospinosulin-1 (**92**) [58].

Kotoku et al. reported that the modifications of **92** structure such as elongating the methyl group close to the furan ring, changing the aromatic ring, and side-chain truncation led to a remarkable loss of selective hypoxia growth inhibition capacity, obviously revealing that the entire structure was substantial for the binding with the target molecule [58]; whilst the analog had a longer side chain partially retained hypoxia-selective inhibitory potential. Therefore, Kotoku et al. synthesized and assessed various furospinosulin-1 (**92**) tail-modified analogs (Scheme 6)

The analog **X** was found to be much more potent than furospinosulin-1 as it possessed hypoxia-selective growth-inhibitory potential (Conc. 1–300 µM) and had powerful in vivo antitumor effectiveness after oral administration (doses 5–25 mg/kg) without side effects [58].

**Scheme 7:**
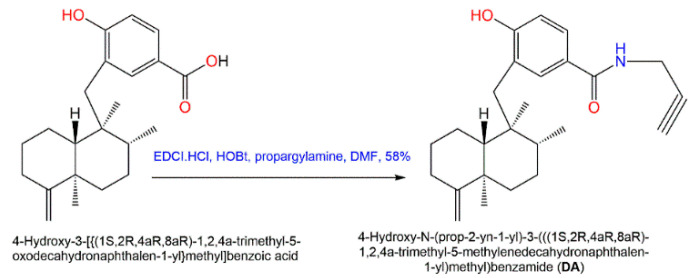
Synthesis of potent structurally modified analog of **52** [47].

Additionally, in 2015, Sumii et al. assessed the structure–activity relationship of (+)-dictyoceratin A (**49**) and (+)-dictyoceratin C (**52**) through the synthesis of structural-modified derivatives of (+)-dictyoceratin C (**52**) and testing their in-vivo antitumor potential [47]. Thus far, the only methyl ester substitution in **52** with propargyl amide (**DA**) had an excellent hypoxia-selective growth prohibition potential at 30 µM relative to **52** (Scheme 7). Thus, it could be efficient for probe molecules synthesis for target identification of **49** and **52** [47].

**Scheme 8:**
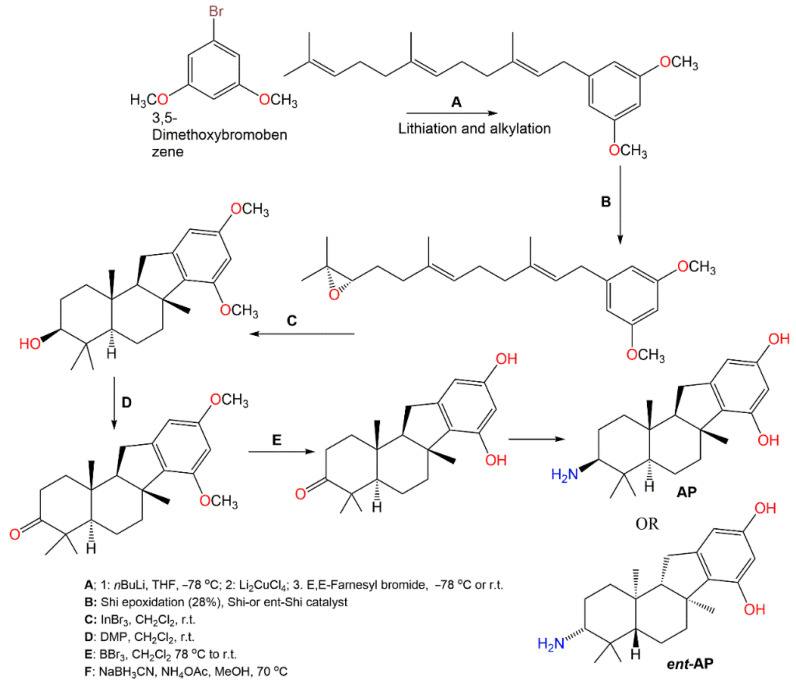
Synthesis of SHIP1-activating analogs of pelorol (**64**) [60].

Pelorol (**64**), sesquiterpene hydroquinone having C8–C21 cyclization had a potent and selective SHIP1 (Src homology 2-containing inositol 5-phosphatase 1) activating potential [59]. However, this compound had catechol moiety that can be either enzymatic or chemically oxidized to orthoquinones, forming covalent linkages with proteins resulting in losing the activity. Additionally, it is highly water-insoluble, which limits its in vivo bioactivity as a SHIP1 activator. Meimetis et al. synthesized more water-soluble amino analogs *ent*-**PA** or (±)-PA (Scheme 8). Synthesis of (±)-PA characterized by generating a tetracyclic ring system through a cation-initiated polyene cyclization [60]. Then, 3,5-Dimethoxybromobenzene lithiation subsequent alkylation with farnesyl bromide produced the 3,5-dimethoxybenzene prenylated intermediate. A racemic epoxide was produced utilizing *m*-chloroperbenzoic acid with subsequent steps giving (±)-PA. Repeating the same procedure using *ent*-Shi catalyst produced *ent*-**PA** analog. These analogs in vitro activated SHIP1, prohibited Akt phosphorylation and had potent in vivo anti-inflammation potential (ED50 0.1 mg/kg, oral gavage) using passive cutaneous anaphylaxis mouse model [60]. The results suggested that *ent*-PA or (±)-**PA** pelorol analogs are promising candidates for further in vivo preclinical investigation as SHIP1-activating therapeutics for treating hematopoietic illnesses, involving aberrant PI3K cell signaling activation.

## 5. Activities of *D. elegans* Extracts and Fractions

BACE1 (β-site of amyloid precursor protein cleaving enzyme) is an enzyme involved in Alzheimer’s disease pathogenesis. The 75% and 100% MeOH C8 fractions of *D. elegans* obtained from the coast of Kauai had significant in vitro BACE1 inhibition (%inhibition 66% and 73%, respectively, at Conc. 30 μg/mL) [61]. Li et al. revealed that the CH_2_Cl_2_/MeOH extract possessed marked IL-6 and TNF-α inhibitory potential [34]. Additionally, the antioxidant potential testing revealed that *D. elegans* hexane extract exhibited significant antioxidant potential in comparison to the ascorbic acid [62]. 

## 6. Conclusions

**Figure 10:**
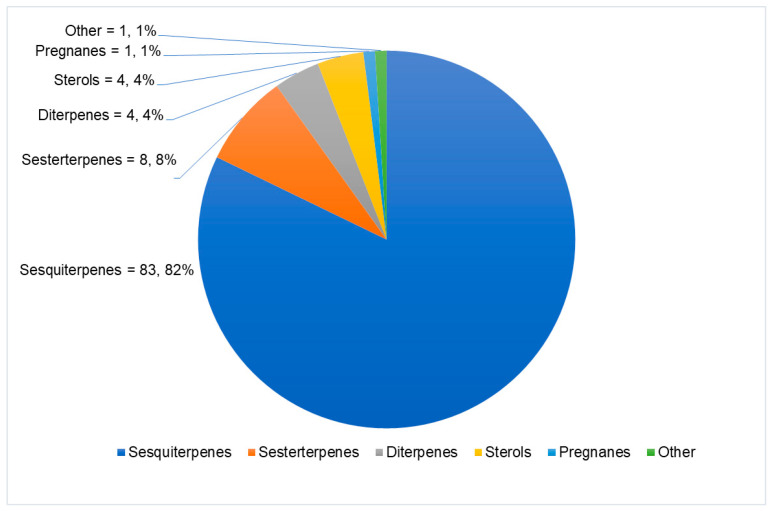
Number and chemical class of reported metabolites from *D. elegans*.

The marine environment is a wealth of biological and chemical diversity. Biometabolites from marine organisms have been proven to be beneficial sources for the discovery of novel drug targets. Among these organisms, sponges are a fascinating marine invertebrate’ phylum, which have been recognized as a big reservoir of biometabolites. The current work highlights one of the most interesting sponge species, *D. elegans*. Over the last 30 years, 101 metabolites have been separated and characterized from *D. elegans*. The results displayed that sesquiterpenes of various classes represented the major metabolites of this sponge. Additionally, few studies reported the isolation of sesterterpenes, sterols, pregnanes, and diterpenes (Figure 10). 

**Figure 11:**
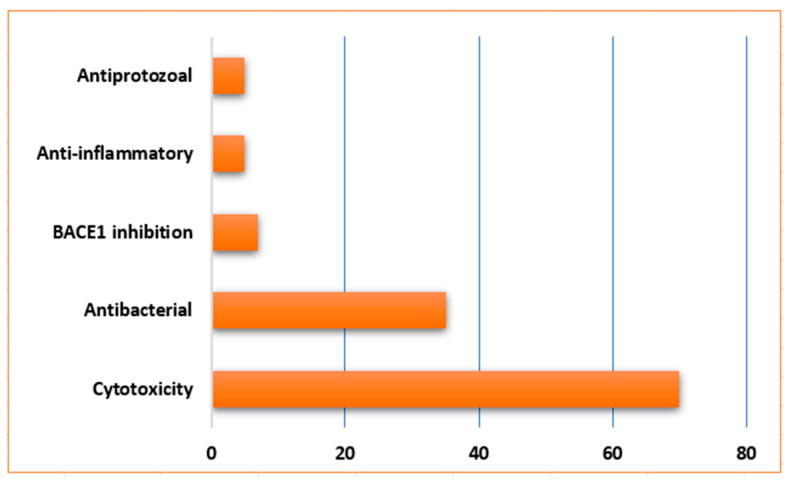
Bioactivities and the number of tested metabolites.

These metabolites have been assessed mainly for their antitumor and antiproliferative potential. Limited studies investigated the antibacterial, anti-inflammatory, β-secretase 1 inhibitory, and antiprotozoal activities (Figure 11) 

Some *D. elegans* sesquiterpenes demonstrated remarkable cytotoxic and antiproliferative activities either in vivo or in vitro towards various cancer cell lines. Further, some sesquiterpenes had promising HIF-1 activating capacity, therefore they could have therapeutic potential as future drug targets for chemotherapeutic drugs, as well as for treating ischemic diseases.

It is noteworthy that limited studies exploring the mechanism of anticancer potential of these metabolites were reported. It was found that these metabolites induced their effectiveness through any of the following mechanisms: hypoxia-targeted growth inhibition (e.g., **48, 49**, and **52**), inhibition of IGF-IR signaling via IGF-2 transcription suppression (e.g., **92**), inhibiting accumulation of HIF-1 and decreasing production of VEGF (e.g., **52**), induction of apoptosis through increasing proteolytic activity of caspases (e.g., **1** and **2**), and cell cycle arrest through boosting p21 expression and suppressing Rb phosphorylation (e.g., **25**). Further, one report revealed the anti-inflammatory potential of **69** through inhibition of IL-6 production.

**Figure 12:**
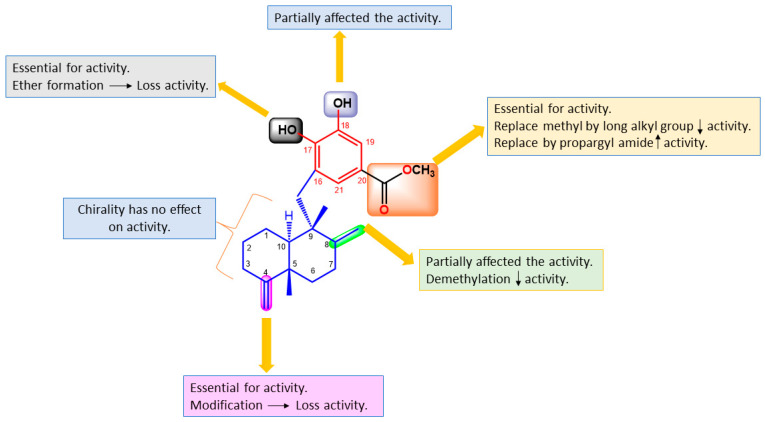
Structural features of sesquiterpene phenols and structure–activity relationship (SAR) hypoxia-selective growth inhibition. Decalin part: blue-colored; P-Hydroxybenzoyl: red-colored; *exo*-olefin: pink-highlighted; Methyl ester: orange-highlighted; 8-Methyl: green-highlighted [24,46,47].

**Figure 13:**
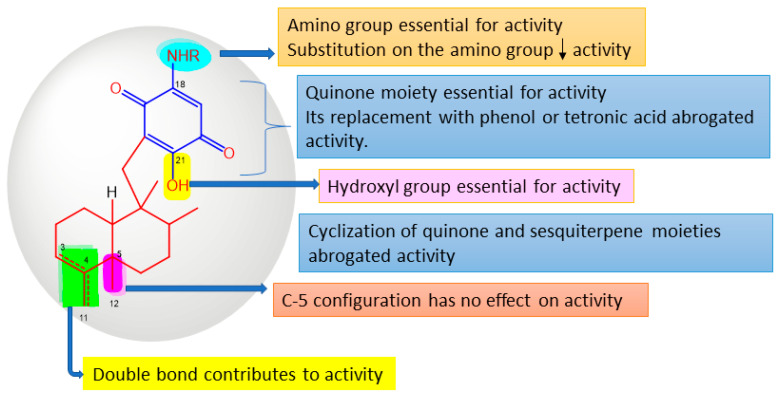
Structural features of sesquiterpene quinones and structure–activity relationship (SAR) for Cytotoxicity and HIF-1 activation effects [16,20,21,22,23]. Decalin part: red-colored; 1,4-Quinone: blue; 5,6-Endocyclic or exocyclic double bond: green-highlighted; 8-Methyl: pink-highlighted; 18-NH: blue-highlighted; 21-OH: yellow-highlighted; ↓: Decrease.

The structure–activity studies demonstrated that the chemical nature of the metabolites’ skeletons, as well as the substituents, were greatly influenced the activities as summarized in Figure 12 and Figure 13. 

On the other side, synthesis studies revealed that the structural modifications and replacement of some functional groups with the more physiologically stable functionalities resulted in more active and useful tags for further functionalization of these metabolites through click chemistry which is a new field for synthesizing drug-like molecules that can accelerate the process of drug discovery. Interestingly, kauamide was proposed to be biosynthesized by cyanobacteria harboring *D. elegans* due to its structure similarity to cyanobacteria reported metabolites, therefore the chemical investigation of symbiotic microorganisms of *D. elegans* could be potential producers of biometabolites originally derived from this sponge. 

Moreover, some bioassays of the reported metabolites revealed no potential effectiveness, providing more potentiality for carrying out other pharmacologic evaluations. Advanced techniques, such as metabolomics, LC/MS/NMR (liquid chromatography/mass spectrometry/nuclear magnetic resonance), and UPLC/MS (Ultra performance liquid chromatography-tandem mass spectrometer) should be employed to explore more bio-metabolites from this sponge. A combination of pathway reconstructing, genetic and enzyme engineering, and metabolic networks could modify this sponge to biosynthesize more novel metabolites having promised structural features or a large quantity of the valuable known ones for pharmaceutical application. Structure–activity relationship studies and chemical syntheses of the promising metabolites give further chances to generate more significant drug leads with optimized chemical stability, activity, and accessibility. Finally, we believed that the metabolites of this sponge deserve much more research attention.

## Figures and Tables

**Figure 8:**
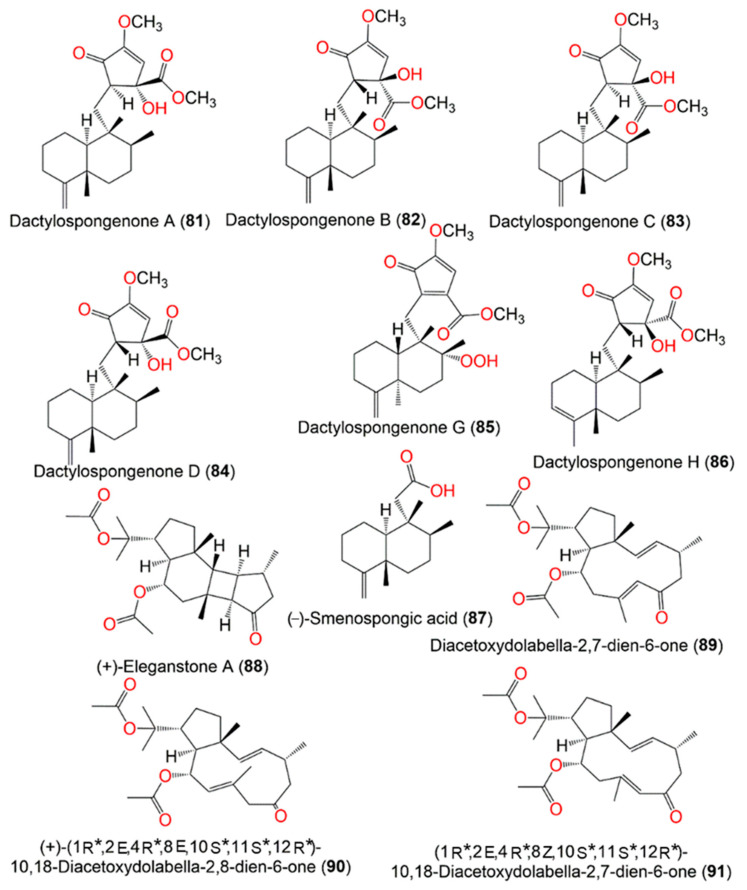
Structures of compounds **81**–**91**.

**Table 1:** Reported secondary metabolites from the four main class of sponges [12].

Sponge Class	Compounds Classes
Calcarea	C_27_ to C_29_ ∆^5,7,9(11),22^ and C_27_ to C_29_ ∆^5,7,22^ sterols Amino alcohols
Hexactinellida	5α(H)-Cholestan-3β-ol/cholest-5-en-3β-ol Ceramide glycosides
Homoscleromorpha	Steroidal alkaloids Peroxy-polyketides
Demospongiae	Pyrroloquinoline, azetidine, pyrrole-2-aminoimidazole, and pentacyclic guanidine alkaloids Norditerpene and norsesterterpene peroxides Tetramic acids Steroidal saponins and glycosides Isomalabaricane triterpenoids Bengamide and bengazoles Hydroxyimino- and 3β-hydroxymethyl-A-nor-sterols 3-Alkylpyridines/3-alkylpiperidines Renieramycins and polyacetylenes Pentacyclic hydroquinones/polyprenylated benzoquinones Adenine- and cyanthiwigin diterpenes Hypotaurocyamine (Sesquiterpene derivatives) Diterpene thio/iso/cyanides and formamides Sesquiterpene thio/iso/cyanides and formamides Aaptamines and bromotyrosines Suberitane-derived sesterterpenes Diterpene, sesquiterpene, and sesterterpenefurans/lactones Scalarane sesterterpenes/sesterterpene hydroquinones Thiazole polyketides Polybrominated diphenyl ethers

∆: Double bond.

**Table 3:** Biological activity of reported metabolites from *Dactylospongia elegans*.

Compound Name	Biological Activity	Assay, Organism, or Cell Line	Biological Results		Ref.
			Compound	Positive Control	
(−)-Ilimaquinone (**1**)	Antitrypanosomal	Semiautomated microdilution/*Trypanosoma brucei*	7.7 µg/mL (IC_50_)	Melarsoprol 0.0026 µg/mL (IC_50_)	[18]
	Antimalarial	Semiautomated microdilution/*P. falciparum* clone K1	1743.0 µg/mL (IC_50_)	Chloroquine 91.0 µg/mL (IC_50_)	[18]
		Semiautomated microdilution/*P. falciparum* clone NF54	949.0 µg/mL (IC_50_)	Chloroquine 4.6 µg/mL (IC_50_)	[18]
	Cytotoxicity	MTT/BC	1.50 µg/mL (IC_50_)	Doxorubicin 0.29 µg/mL (IC_50_)	[21]
		MTT/NCI-H187	3.37 µg/mL (IC_50_)	Doxorubicin 0.06 µg/mL (IC_50_)	[21]
		SRB/SF-268	2.7 µM (GI_50_)	Vehicle-DMSO	[22]
		SRB/MCF-7	3.9 µM (GI_50_)	Vehicle -DMSO	[22]
		SRB/H460	1.8 µM (GI_50_)	Vehicle -DMSO	[22]
		SRB/HT-29	5.4 µM (GI_50_)	Vehicle-DMSO	[22]
		SRB/CHO-K1	2.0 µM (GI_50_)	Vehicle-DMSO	[22]
	β-Secretase 1 inhibition	BACE1	65.0 µM (IC_50_)	-	[26]
	Cytotoxicity	MTT/U251	19.3 µM (CC_50_)	Vehicle-DMSO	[26]
		MTT/Panc-1	20.4 µM (CC_50_)	Vehicle-DMSO	[26]
	Antibacterial	Broth microdilution/*S. aureus* USA300 LAC	5.6 µg/mL (MIC)	Vancomycin 1.0 µg/mL (MIC)	[27]
		Broth microdilution/*S. pyogenes* ATCC 12344	2.8 µg/mL (MIC)	Vancomycin 0.25 µg/mL (MIC)	[27]
		Broth microdilution/*E. faecium* Efm-HS0649	11.2 µg/mL (MIC)	Vancomycin ˃ 64.0 µg/mL (MIC)	[27]
5-(+)-*Epi*-Ilimaquinone (**2**)	Cytotoxicity	A549	0.9 µg/mL (IC_50_)	-	[16]
		HT-29	3.4 µg/mL (IC_50_)	-	[16]
		B_16_F_10_	1.1 µg/mL (IC_50_)	-	[16]
		P388	2.2 µg/mL (IC_50_)	-	[16]
	Cytotoxicity	MTT/L5178Y	2.23 µM (IC_50_)	Kahalalide F 4.30 µM (IC_50_)	[28]
	Antibacterial	Broth microdilution/*S. aureus* ATCC 25923	50.0 µM (MIC)	Moxifloxacin 3.89 µM (MIC)	[28]
		Broth microdilution/*S. aureus* ATCC 700699	50.0 µM (MIC)	Moxifloxacin 3.89 µM (MIC)	[28]
	Cytotoxicity	MTT/U251	19.4 µM (CC_50_)	Vehicle-DMSO	[26]
		MTT/Panc-1	16.2 µM (CC_50_)	Vehicle-DMSO	[26]
	Antibacterial	Broth microdilution/*S. aureus* USA300 LAC	5.6 µg/mL (MIC)	Vancomycin 1.0 µg/mL (MIC)	[27]
		Broth microdilution/*S. pyogenes* ATCC 12344	2.8 µg/mL (MIC)	Vancomycin 0.25 µg/mL (MIC)	[27]
		Broth microdilution/*E. faecium* Efm-HS0649	11.2 µg/mL (MIC)	Vancomycin ˃ 64.0 µg/mL (MIC)	[27]
(−)-Dactyloquinone A (**4**)	Antibacterial	Broth microdilution/*S. pyogenes* ATCC 12344	44.5 µg/mL (MIC)	Vancomycin 0.25 µg/mL (MIC)	[27]
		Broth microdilution/*E. faecium* Efm-HS0649	22.2 µg/mL (MIC)	Vancomycin ˃ 64.0 µg/mL (MIC)	[27]
(−)-Dactyloquinone B (**5**)	Cytotoxicity	SRB/SF-268	32.0 µM (GI_50_)	Vehicle -DMSO	[22]
		SRB/MCF-7	41.0 µM (GI_50_)	Vehicle -DMSO	[22]
		SRB/H460	30.0 µM (GI_50_)	Vehicle -DMSO	[22]
		SRB/HT-29	46.0 µM (GI_50_)	Vehicle -DMSO	[22]
		SRB/CHO-K1	43.0 µM (GI_50_)	Vehicle -DMSO	[22]
	Antibacterial	Broth microdilution/*S. aureus* USA300 LAC	178.0 µg/mL (MIC)	Vancomycin 1.0 µg/mL (MIC)	[27]
		Broth microdilution/*S. pyogenes* ATCC 12344	22.2 µg/mL (MIC)	Vancomycin 0.25 µg/mL (MIC)	[27]
		Broth microdilution/*E. faecium* Efm-HS0649	22.2 µg/mL (MIC)	Vancomycin ˃ 64.0 µg/mL (MIC)	[27]
(−)-Dactyloquinone C (**7**)	Antibacterial	Broth microdilution/*S. aureus* USA300 LAC	11.1 µg/mL (MIC)	Vancomycin 1.0 µg/mL (MIC)	[27]
		Broth microdilution/*S. pyogenes* ATCC 12344	5.6 µg/mL (MIC)	Vancomycin 0.25 µg/mL (MIC)	[27]
		Broth microdilution/*E. faecium* Efm-HS0649	5.6 µg/mL (MIC)	Vancomycin ˃ 64.0 µg/mL (MIC)	[27]
(−)-Dactyloquinone D (**8**)	Antibacterial	Broth microdilution/*S. pyogenes* ATCC 12344	89.0 µg/mL (MIC)	Vancomycin 0.25 µg/mL (MIC)	[27]
		Broth microdilution/*E. faecium* Efm-HS0649	178.0 µg/mL (MIC)	Vancomycin ˃ 64.0 µg/mL (MIC)	[27]
(+)-Dactyloquinone E (**9**)	Antibacterial	Broth microdilution/*S. pyogenes* ATCC 12344	22.2 µg/mL (MIC)	Vancomycin 0.25 µg/mL (MIC)	[27]
		Broth microdilution/*E. faecium* Efm-HS0649	178.0 µg/mL (MIC)	Vancomycin ˃ 64.0 µg/mL (MIC)	[27]
(+)-Isospongiaquinone (**11**)	Cytotoxicity	MTT/L5178Y	1.34 µM (IC_50_)	Kahalalide F 4.30 µM (IC_50_)	[28]
	Antibacterial	Broth microdilution/*S. aureus* ATCC 25923	50.0 µM (MIC)	Moxifloxacin 3.89 µM (MIC)	[28]
		Broth microdilution/*S. aureus* ATCC 700699	50.0 µM (MIC)	Moxifloxacin 3.89 µM (MIC)	[28]
		Broth microdilution/*E. faecalis* ATCC 51299	50.0 µM (MIC)	Ciprofloxacin 0.02 µM (MIC)	[28]
		Broth microdilution/*E. faecalis* ATCC 35677	50.0 µM (MIC)	Ciprofloxacin 0.02 µM (MIC)	[28]
		Broth microdilution/*E. faecalis* ATCC 700221	50.0 µM (MIC)	Ciprofloxacin 0.02 µM (MIC)	[28]
Mamanuthaquinone (**14**)	Cytotoxicity	MTT/BC	2.61 µg/mL (IC_50_)	Doxorubicin 0.29 µg/mL (IC_50_)	[21]
		MTT/NCI-H187	8.78 µg/mL (IC_50_)	Doxorubicin 0.06 µg/mL (IC_50_)	[21]
Hyatellaquinone (**15**)	Cytotoxicity	MTT/BC	4.45 µg/mL (IC_50_)	Doxorubicin 0.29 µg/mL (IC_50_)	[21]
		MTT/NCI-H187	10.90 µg/mL (IC_50_)	Doxorubicin 0.06 µg/mL (IC_50_)	[21]
		MTT/BC	1.50 µg/mL (IC_50_)	Doxorubicin 0.29 µg/mL (IC_50_)	[21]
(+)-Isohyatellaquinone (**16**)	Cytotoxicity	MTT/BC	6.69 µg/mL (IC_50_)	Doxorubicin 0.29 µg/mL (IC_50_)	[21]
		MTT/NCI-H187	11.52 µg/mL (IC_50_)	Doxorubicin 0.06 µg/mL (IC_50_)	[21]
Neomamanuthaquinone (**18**)	Cytotoxicity	MTT/BC	8.42 µg/mL (IC_50_)	Doxorubicin 0.29 µg/mL (IC_50_)	[21]
9-*Epi*-7,8-Dehydrocyclospongiaquinone-2 (**20**)	Cytotoxicity	MTT/BC	7.38 µg/mL (IC_50_)	Doxorubicin 0.29 µg/mL (IC_50_)	[21]
		MTT/NCI-H187	12.40 µg/mL (IC_50_)	Doxorubicin 0.06 µg/mL (IC_50_)	[21]
Smenospongine (**25**)	Cytotoxicity	A549	5.7 µg/mL (IC_50_)	-	[16]
		HT-29	4.0 µg/mL (IC_50_)	-	[16]
		B_16_F_10_	4.1 µg/mL (IC_50_)	-	[16]
		P388	2.6 µg/mL (IC_50_)	-	[16]
		MTT/U251	2.4 µM (CC_50_)	Vehicle-DMSO	[26]
	β-Secretase 1 inhibition	BACE1	65.0 µM (IC_50_)	-	[26]
Smenospongimine (**27**)	Cytotoxicity	CCK-8/DU145	3.5 µM (IC_50_)	Cisplatin 2.9 µM (IC_50_)	[32]
		CCK-8/SW1990	4.2 µM (IC_50_)	Cisplatin 1.2 µM (IC_50_)	[32]
		CCK-8/Huh7	2.3 µM (IC_50_)	Cisplatin 2.2 µM (IC_50_)	[32]
		CCK-8/Panc-1	5.8 µM (IC_50_)	Cisplatin 4.6 µM (IC_50_)	[32]
Smenospongine B (**28**)	Cytotoxicity	SRB/SF-268	9.7 µM (GI_50_)	Vehicle-DMSO	[22]
		SRB/MCF-7	10.0 µM (GI_50_)	Vehicle-DMSO	[22]
		SRB/H460	6.0 µM (GI_50_)	Vehicle-DMSO	[22]
		SRB/HT-29	6.0 µM (GI_50_)	Vehicle-DMSO	[22]
		SRB/CHO-K1	3.0 µM (GI_50_)	Vehicle-DMSO	[22]
Smenospongine C (**29**)	Cytotoxicity	SRB/SF-268	20.0 µM (GI_50_)	Vehicle-DMSO	[22]
		SRB/MCF-7	31.0 µM (GI_50_)	Vehicle-DMSO	[22]
		SRB/H460	14.0 µM (GI_50_)	Vehicle-DMSO	[22]
		SRB/HT-29	28.0 µM (GI_50_)	Vehicle-DMSO	[22]
		SRB/CHO-K1	18.0 µM (GI_50_)	Vehicle-DMSO	[22]
	Antibacterial	Broth microdilution/*S. aureus* ATCC 25923	50.0 µM (MIC)	Moxifloxacin 3.89 µM (MIC)	[28]
		Broth microdilution/*S. aureus* ATCC 700699	50.0 µM (MIC)	Moxifloxacin 3.89 µM (MIC)	[28]
Smenospongorine (**30**)	Cytotoxicity	MTT/U251	19.4 µM (CC_50_)	Vehicle-DMSO	[26]
		MTT/Panc-1	22.6 µM (CC_50_)	Vehicle-DMSO	[26]
		CCK-8/DU145	4.2 µM (IC_50_)	Cisplatin 2.9 µM (IC_50_)	[32]
		CCK-8/SW1990	4.4 µM (IC_50_)	Cisplatin 1.2 µM (IC_50_)	[32]
		CCK-8/Huh7	3.0 µM (IC_50_)	Cisplatin 2.2 µM (IC_50_)	[32]
		CCK-8/Panc-1	7.7 µM (IC_50_)	Cisplatin 4.6 µM (IC_50_)	[32]
Smenospongiarine (**32**)	Cytotoxicity	MTT/U251	4.5 µM (CC_50_)	Vehicle-DMSO	[26]
		MTT/Panc-1	15.1 µM (CC_50_)	Vehicle-DMSO	[26]
		CCK-8/DU145	6.1 µM (IC_50_)	Cisplatin 2.9 µM (IC_50_)	[32]
		CCK-8/SW1990	5.9 µM (IC_50_)	Cisplatin 1.2 µM (IC_50_)	[32]
		CCK-8/Huh7	3.7 µM (IC_50_)	Cisplatin 2.2 µM (IC_50_)	[32]
		CCK-8/Panc-1	8.7 µM (IC_50_)	Cisplatin 4.6 µM (IC_50_)	[32]
5-(+)-*Epi*-Smenospongiarine (33)	Cytotoxicity	A549	0.8 µg/mL (IC_50_)	-	[16]
		HT-29	0.9 µg/mL (IC_50_)	-	[16]
		B_16_F_10_	0.6 µg/mL (IC_50_)	-	[16]
		P388	0.7 µg/mL (IC_50_)	-	[16]
Smenospongidine (**35**)	Cytotoxicity	MTT/U251	4.0 µM (CC_50_)	Vehicle-DMSO	[26]
		MTT/Panc-1	12.6 µM (CC_50_)	Vehicle-DMSO	[26]
5-(+)-*Epi*-Smenospongidine (**36**)	Cytotoxicity	A549	3.9 µg/mL (IC_50_)	-	[16]
		HT-29	2.4 µg/mL (IC_50_)	-	[16]
		B_16_F_10_	1.9 µg/mL (IC_50_)	-	[16]
		P388	1.9 µg/mL (IC_50_)	-	[16]
		MTT/L5178Y	1.34 µM (IC_50_)	Kahalalide F 4.30 µM (IC_50_)	[28]
	Antibacterial	Broth microdilution/*S. aureus* ATCC 25923	50.0 µM (MIC)	Moxifloxacin 3.89 µM (MIC)	[28]
		Broth microdilution/*E. faecalis* ATCC 35677	50.0 µM (MIC)	Ciprofloxacin 0.02 µM (MIC)	[28]
		Broth microdilution/*E. faecalis* ATCC 700221	25.0 µM (MIC)	Ciprofloxacin 0.02 µM (MIC)	[28]
Isosmenospongine (**39**)	Cytotoxicity	MTT/L5178Y	1.69 µM (IC_50_)	Kahalalide F 4.30 µM (IC_50_)	[28]
	Antibacterial	Broth microdilution/*S. aureus* ATCC 25923	25.0 µM (MIC)	Moxifloxacin 3.89 µM (MIC)	[28]
		Broth microdilution/*S. aureus* ATCC 700699	12.5 µM (MIC)	Moxifloxacin 3.89 µM (MIC)	[28]
		Broth microdilution/*E. faecalis* ATCC 29212	25.0 µM (MIC)	Ciprofloxacin 0.02 µM (MIC)	[28]
		Broth microdilution/*E. faecalis* ATCC 51299	25.0 µM (MIC)	Ciprofloxacin 0.02 µM (MIC)	[28]
		Broth microdilution/*E. faecalis* ATCC 35677	25.0 µM (MIC)	Ciprofloxacin 0.02 µM (MIC)	[28]
		Broth microdilution/*E. faecalis* ATCC 700221	25.0 µM (MIC)	Ciprofloxacin 0.02 µM (MIC)	[28]
Nakijiquinone A (**40**)	Cytotoxicity	MTT/L5178Y	6.48 µM (IC_50_)	Kahalalide F 4.30 µM (IC_50_)	[28]
	Antibacterial	Broth microdilution/*S. aureus* ATCC 700699	50.0 µM (MIC)	Moxifloxacin 3.89 µM (MIC)	[28]
		Broth microdilution/*E. faecalis* ATCC 51299	50.0 µM (MIC)	Ciprofloxacin 0.02 µM (MIC)	[28]
Nakijiquinone B (**41**)	Antibacterial	Broth microdilution/*S. aureus* ATCC 25923	50.0 µM (MIC)	Moxifloxacin 3.89 µM (MIC)	[28]
Nakijiquinone G (**43**)	Cytotoxicity	MTT/L5178Y	2.74 µM (IC_50_)	Kahalalide F 4.30 µM (IC_50_)	[28]
5-*Epi*-Nakijiquinone Q (**44**)	Antibacterial	Broth microdilution/*S. aureus* ATCC 25923	25.0 µM (MIC)	Moxifloxacin 3.89 µM (MIC)	[28]
		Broth microdilution/*S. aureus* ATCC 700699	50.0 µM (MIC)	Moxifloxacin 3.89 µM (MIC)	[28]
		Broth microdilution/*E. faecalis* ATCC 35667	50.0 µM (MIC)	Ciprofloxacin 0.02 µM (MIC)	[28]
(+)-Dictyoceratin A (**49**)	Cytotoxicity	MTT/U251	2.8 µM (CC_50_)	Vehicle-DMSO	[26]
		MTT/Panc-1	21.7 µM (CC_50_)	Vehicle-DMSO	[26]
	Antibacterial	Broth microdilution/*S. aureus* USA300 LAC	2.9 µg/mL (MIC)	Vancomycin 1.0 µg/mL (MIC)	[27]
		Broth microdilution/*S. pyogenes* ATCC 12344	2.9 µg/mL (MIC)	Vancomycin 0.25 µg/mL (MIC)	[27]
		Broth microdilution/*E. faecium* Efm-HS0649	1.4 µg/mL (MIC)	Vancomycin ˃64.0 µg/mL (MIC)	[27]
(+)-19-Methoxy-dictyoceratin-A (**50**)	Cytotoxicity	CCK-8/DU145	24.4 µM (IC_50_)	Cisplatin 2.9 µM (IC_50_)	[32]
		CCK-8/SW1990	21.4 µM (IC_50_)	Cisplatin 1.2 µM (IC_50_)	[32]
		CCK-8/Huh7	17.4 µM (IC_50_)	Cisplatin 2.2 µM (IC_50_)	[32]
		CCK-8/Panc-1	37.8 µM (IC_50_)	Cisplatin 4.6 µM (IC_50_)	[32]
(+)-Dictyoceratin B (**51**)	Cytotoxicity	MTT/U251	8.4 µM (CC_50_)	Vehicle-DMSO	[26]
		MTT/Panc-1	54.6 µM (CC_50_)	Vehicle-DMSO	[26]
	Antibacterial	Broth microdilution/*S. aureus* USA300 LAC	12.1 µg/mL (MIC)	Vancomycin 1.0 µg/mL (MIC)	[27]
		Broth microdilution/*S. pyogenes* ATCC 12344	1.5 µg/mL (MIC)	Vancomycin 0.25 µg/mL (MIC)	[27]
		Broth microdilution/*E. faecium* Efm-HS0649	3.0 µg/mL (MIC)	Vancomycin ˃ 64.0 µg/mL (MIC)	[27]
(+)-Dictyoceratin C (**52**)	Cytotoxicity	MTT/U251	4.1 µM (CC_50_)	Vehicle-DMSO	[26]
		MTT/Panc-1	88.9 µM (CC_50_)	Vehicle-DMSO	[26]
		CCK-8/DU145	8.3 µM (IC_50_)	Cisplatin 2.9 µM (IC_50_)	[32]
		CCK-8/SW1990	7.9 µM (IC_50_)	Cisplatin 1.2 µM (IC_50_)	[32]
		CCK-8/Huh7	6.9 µM (IC_50_)	Cisplatin 2.2 µM (IC_50_)	[32]
		CCK-8/Panc-1	9.2 µM (IC_50_)	Cisplatin 4.6 µM (IC_50_)	[32]
(−)-Xishaeleganin C (**54**)	Antibacterial	Broth microdilution/*S. aureus* USA300 LAC	11.1 µg/mL (MIC)	Vancomycin 1.0 µg/mL (MIC)	[27]
		Broth microdilution/*S. pyogenes* ATCC 12344	2.8 µg/mL (MIC)	Vancomycin 0.25 µg/mL (MIC)	[27]
		Broth microdilution/*E. faecium* Efm-HS0649	5.6 µg/mL (MIC)	Vancomycin ˃64.0 µg/mL (MIC)	[27]
(+)-Xishaeleganin D (**55**)	Antibacterial	Broth microdilution/*S. pyogenes* ATCC 12344	11.6 µg/mL (MIC)	Vancomycin 0.25 µg/mL (MIC)	[27]
(−)-Xishaeleganin B (**57**)	Antibacterial	Broth microdilution/*S. aureus* USA300 LAC	1.5 µg/mL (MIC)	Vancomycin 1.0 µg/mL (MIC)	[27]
		Broth microdilution/*S. pyogenes* ATCC 12344	1.5 µg/mL (MIC)	Vancomycin 0.25 µg/mL (MIC)	[27]
		Broth microdilution/*E. faecium* Efm-HS0649	3.0 µg/mL (MIC)	Vancomycin ˃64.0 µg/mL (MIC)	[27]
Pelorol (**64**)	Antitrypanosomal	Semiautomated microdilution/*Trypanosoma brucei*	17.4 µg/mL (IC_50_)	Melarsoprol 0.0026 µg/mL (IC_50_)	[18]
	Antimalarial	Semiautomated microdilution/*P. falciparum* clone K1	786.0 µg/mL (IC_50_)	Chloroquine 91.0 µg/mL (IC_50_)	[18]
		Semiautomated microdilution/*P. falciparum* clone NF54	1911.0 µg/mL (IC_50_)	Chloroquine 4.6µg/mL (IC_50_)	[18]
	Antibacterial	Broth microdilution/*S. aureus* ATCC 25923	6.25 µM (MIC)	Moxifloxacin 3.89 µM (MIC)	[28]
		Broth microdilution/*S. aureus* ATCC 700699	3.125 µM (MIC)	Moxifloxacin 3.89 µM (MIC)	[28]
		Broth microdilution/*E. faecalis* ATCC 29212	12.5 µM (MIC)	Ciprofloxacin 0.02 µM (MIC)	[28]
		Broth microdilution/*E. faecalis* ATCC 51299	12.5 µM (MIC)	Ciprofloxacin 0.02 µM (MIC)	[28]
		Broth microdilution/*E. faecalis* ATCC 35677	25.0 µM (MIC)	Ciprofloxacin 0.02 µM (MIC)	[28]
		Broth microdilution/*E. faecalis* ATCC 700221	12.5 µM (MIC)	Ciprofloxacin 0.02 µM (MIC)	[28]
Nakijinol B (**65**)	Cytotoxicity	SRB/SF-268	24.0 µM (GI_50_)	Vehicle -DMSO	[22]
		SRB/MCF-7	35.0 µM (GI_50_)	Vehicle -DMSO	[22]
		SRB/H460	24.0 µM (GI_50_)	Vehicle -DMSO	[22]
		SRB/HT-29	21.0 µM (GI_50_)	Vehicle -DMSO	[22]
		SRB/CHO-K1	11.0 µM (GI_50_)	Vehicle -DMSO	[22]
(−)-Dactyltronic acid A (**71**)	Antibacterial	Broth microdilution/*Vibrio* *parahemolyticus*	3.45 µM (MIC)	Ciprofloxacin 1.25 µM (MIC)	[33]
(−)-Dactyltronic acid B (**72**)	Antibacterial	Broth microdilution/*Vibrio* *parahemolyticus*	3.45 µM (MIC)	Ciprofloxacin 1.25 µM (MIC)	[33]
(+)-Dactylospene B (**77**)	Anti-inflammatory	Griess reagent/LPS	77.5% NO inhibition	-	[35]
(+)-Dactylospene C (**78**)	Cytotoxicity	CCK-8/DU145	13.35 µM (IC_50_)	Cisplatin 2.90 µM (IC_50_)	[35]
		CCK-8/SW1990	7.40 µM (IC_50_)	Cisplatin 5.09 µM (IC_50_)	[35]
		CCK-8/Huh7	2.37 µM (IC_50_)	Cisplatin 1.11 µM (IC_50_)	[35]
	Anti-inflammatory	Griess reagent/LPS	77.5% NO inhibition	-	[35]
Dactylospongenone A (**81**)	Cytotoxicity	B_16_F_10_	2.1 µg/mL (IC_50_)	-	[16]
		P388	0.6 µg/mL (IC_50_)	-	[16]
(−)-Luffariellolide (**94**)	Cytotoxicity	CCK-8/DU145	3.21 µM (IC_50_)	Cisplatin 2.90 µM (IC_50_)	[35]
		CCK-8/SW1990	3.55 µM (IC_50_)	Cisplatin 5.09 µM (IC_50_)	[35]
		CCK-8/Huh7	3.61 µM (IC_50_)	Cisplatin 1.11 µM (IC_50_)	[35]
		CCK-8/Panc-1	5.21 µM (IC_50_)	Cisplatin 4.59 µM (IC_50_)	[35]
(−)-Dactylospene A (**95**)	Cytotoxicity	CCK-8/DU145	2.87 µM (IC_50_)	Cisplatin 2.90 µM (IC_50_)	[35]
		CCK-8/SW1990	2.11 µM (IC_50_)	Cisplatin 5.09 µM (IC_50_)	[35]
		CCK-8/Huh7	2.87 µM (IC_50_)	Cisplatin 1.11 µM (IC_50_)	[35]
		CCK-8/Panc-1	7.59 µM (IC_50_)	Cisplatin 4.59 µM (IC_50_)	[35]
Pregna-1,20-dien-3-one (**96**)	Antibacterial	Broth microdilution/*B. cereus*	4.19 µM (MIC)	Ciprofloxacin 1.25 µM (MIC)	[33]

## Data Availability

Not applicable.

## Abbreviations

A549: Lung adenocarcinoma epithelial cell line; A375: Human melanoma cell line; L5178Y: Mouse lymphoma cell line; ATRA: all-trans-retinoic acid; B_16_F_10_: Human murine melanoma cell; BACE1: Fluorescent beta secretase assay kits; BC: Human breast cancer cell line; CCK-8: Cell Counting Kit-8; BCR-ABL: Mutation that is formed by the combination of two genes; BMT: Bone marrow transplant; C_50_: Cytotoxicity concentration 50; CH_2_Cl_2_: Dichloromethane; CIP/KIP: Family cyclin-dependent kinase; CML: Chronic myelogenous leukemia; Crkl: Adaptor protein; DU145: Human prostate cancer cell line; ECD: Electronic circular dichroism; Et_2_O: Diethyl ether; EtOAc: Ethyl acetate; EtOH: Ethanol; GI_50_: Growth inhibitory power of the test agent; H_2_O: Water; HIF-1: Hypoxia-inducible factor-1; HL60: Human acute promyelocytic leukemia cell line; HT-29: Human colon adenocarcinoma cell line; Huh7: Human liver cancer cell lines; HepG2: Human hepatocarcinoma cell line; HPLC: High pressure liquid chromatography; HUVEC; Human umbilical vein endothelial cells; IGF-2: Insulin-like growth factor-2 gene; IC_50_: Concentration causing 50% growth inhibition; LEDGF/p75: Lens epithelium-derived growth factor p75: LPS: Lipopolysaccharide; K562: Human chronic myelogenous leukemia cell line; LC/MS/NMR: Liquid chromatography/mass spectrometry/nuclear magnetic resonance; MCF-7: Human breast cancer cell line; MDA-MB-231: Epithelial, human breast cancer cell line; MeOH: Methanol; MTT: 3-(4,5-Dimethylthiazol-2-yl)-2,5-diphenyl tetrazolium bromide; NCI-H187: Human small cell lung cancer cell line; NMR: Nuclear magnetic resonance; NOESY: Nuclear Overhauser Effect Spectroscopy; NO: Nitrous oxide; RP-18: Reversed-phase C_18_ silica gel; Panc-1: Human pancreatic carcinoma cell lines; P3: Human epithelial teratocarcinoma cells; P21: Potent cyclin-dependent kinase inhibitor; P388: Leukemia cell line; PC-3: Human prostatic cancer cell line; PC-9: Human adenocarcinoma cell line: p54^nrb^: Non-POU domain-containing octamer-binding protein; Ph: Philadelphia chromosome; SRB: Sulfurhodamine B; SiO_2_ CC: Silica gel column chromatography; Sp1: Proximal specificity protein 1; SW1990: Human pancreatic cancer cell line; T-47D: Human breast cancer cell line; THP-1: Human acute monocytic leukemia cell line; U251: Human glioma cell lines; U937: Human histiocytic lymphoma cell line; UPLC/MS: Ultra performance liquid chromatography-tandem mass spectrometer; VEGF: Vascular endothelial growth factor: WST-8: Colorimetric Cell Viability Kit; WST-8: Water-soluble tetrazolium salt-8.
